# Supplementary material for: Uncovering the signaling landscape controlling breast cancer cell migration identifies novel metastasis driver genes
Source: Nat Commun. 2019 Jul 5;10:2983. doi: 10.1038/s41467-019-11020-3 (PMC6611796; doi:10.1038/s41467-019-11020-3)
Supplement: Supplementary file 1 — Supplementary Information [file 41467_2019_11020_MOESM1_ESM.pdf]

Supplementary Information

Uncovering the signaling landscape controlling breast cancer cell migration identifies novel metastasis driver genes

Koedoot et al.

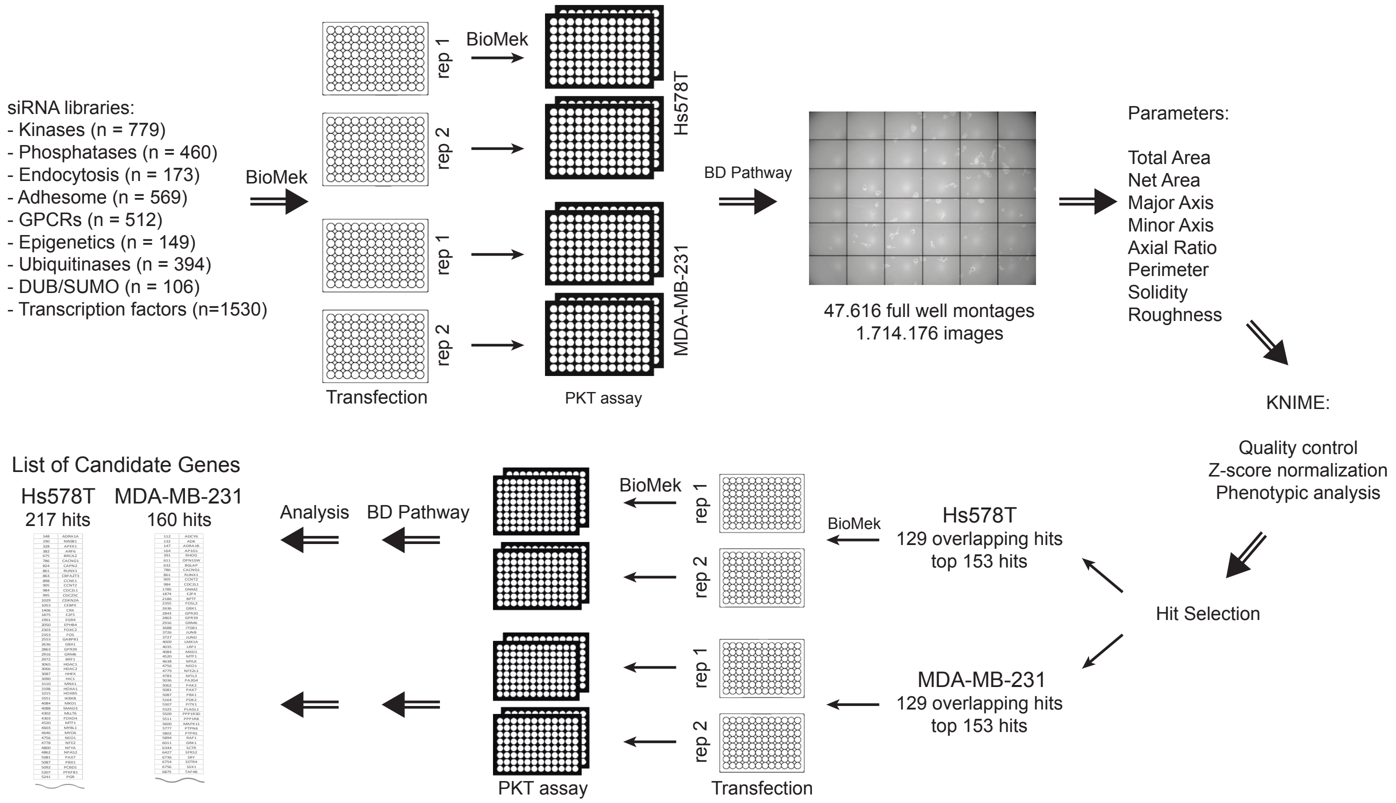

Supplementary Figure 1. ***RNAi PKT screen setup.*** Transfection of up to 10 siRNA library plates per run was performed by automated liquid handling (BioMek). Transfections were performed in duplicate, on different days with separately grown cell cultures. Transfected cells were washed with PBS, trypsinized, diluted and resus-pended into single cell suspension, before being seeded in duplicate PKT assay plates (technical replicate). All steps were optimized for automated liquid handling. Whole well montages (6x6) were acquired on a BD Path-way BioImager using transmitted light, and a robotic arm (Twister II, Caliper) placed and removed the PKT assay plates on the microscope. PKT images were analyzed using PhagoTracker software as described previ-ously (32, 33). Quantitative output was normalized to mock control (robust Z-score) using KNIME. Visual inspection of images led to the identification of migratory phenotypes and corresponding Z-scores were assigned (cut-off Z-scores can be found in the methods section). The selected migratory phenotypes were subsequently used for supervised clustering of hits by means of principal component analysis and plotted in a 3D phenotypic space (Fig. 1E,F). Primary hits were selected in two ways: hits that showed overlap between the two cell lines for each migratory phenotype (129 hits) and the top hits affecting cell migration within each cell line (153 hits in Hs578T, 153 hits in MDA-MB-231). Primary hits were validated by deconvolution screens, eval-uating the effect of SMARTpool and single siRNA sequences in PKT assays as before. Hits were considered validated if the SMARTpool showed consistent results and at least 2 of 4 single siRNA sequences showed the same phenotype. Ultimately, 217 hits were validated in the Hs578T cells and 160 hits in the MDA-MB-231.

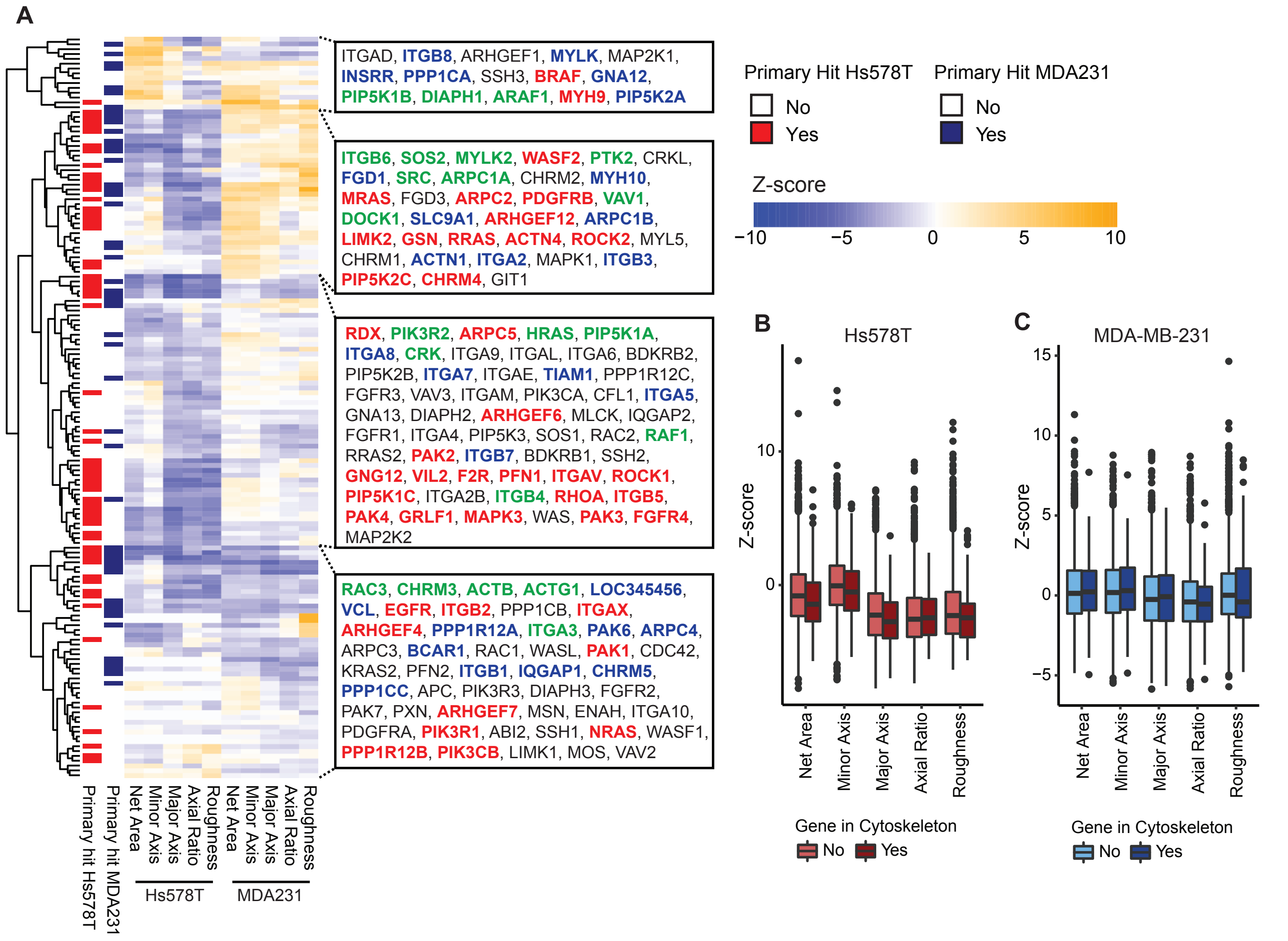

Supplementary Figure 2. ***Cytoskeletal genes in the primary PKT screen.*** (A) Primary screen Z-scores of genes in the KEGG pathway "Regulation of the actin cytoskeleton". Red = hit in Hs578T, blue = hit in MDA-MB-231, green = hit in both cell lines. (B) Z-score distribution of genes that are in the "Regulation of the actin cytoskeleton" pathway or other genes for Hs578T. Boxplots represent median, first and third quartile. (C) Same as in B, but for MDA-MB-231. Shown Z-scores are averages of technical and biological duplicates.

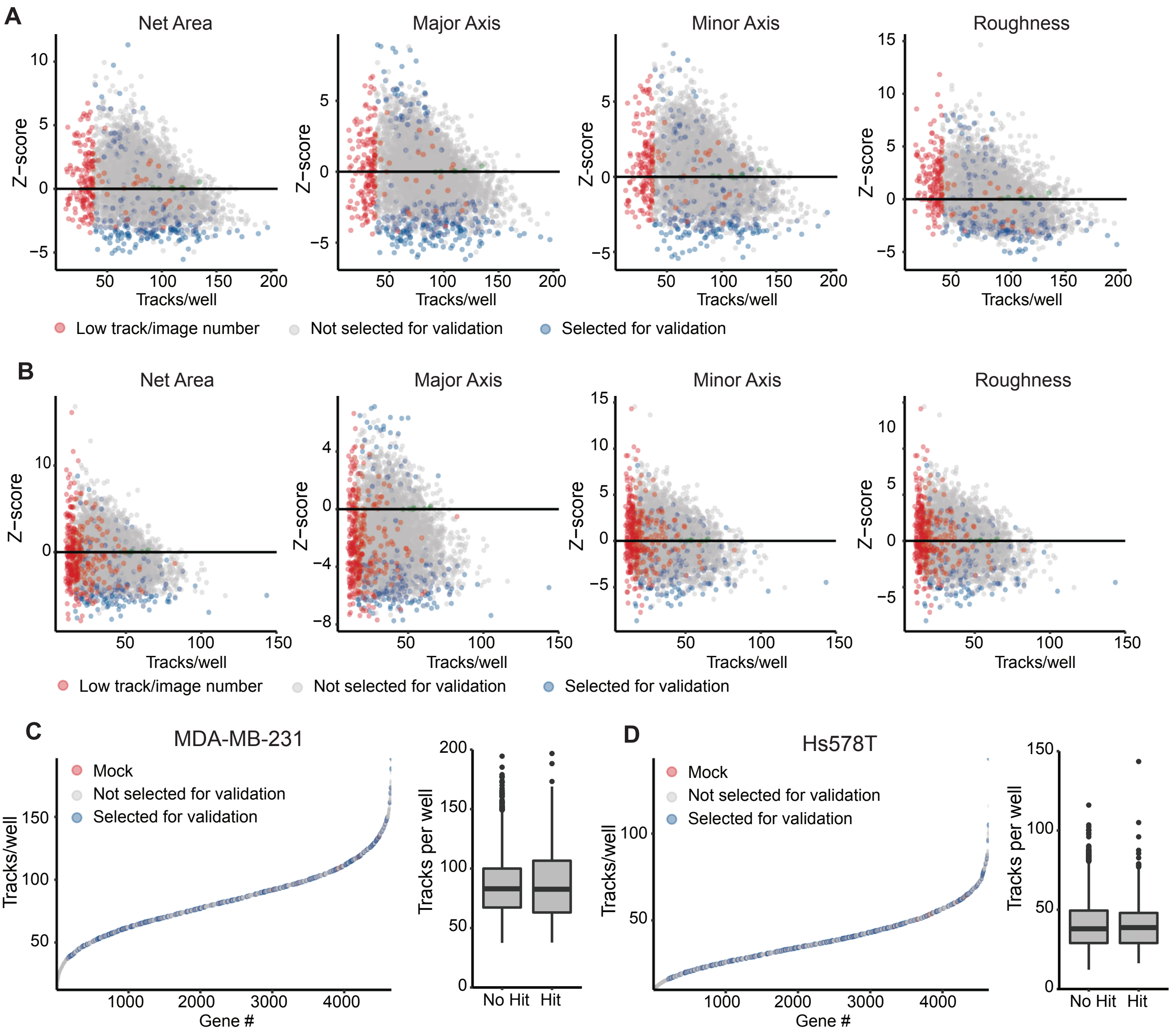

Supplementary Figure 3. ***Proliferative effects of migration candidates primary PKT screen.*** (A) Relation between track parameter Z-scores and number of tracks imaged per well in MDA-MB-231. (B) Relation between track parameter Z-scores and number of tracks imaged per well in Hs578T. (C) Comparison of number of tracks for non-hits and hits in MDA-MB-231. (D) Comparison of number of tracks for non-hits and hits of the primary screen in Hs578T. Shown Z-scores are averages of technical and biological duplicates.

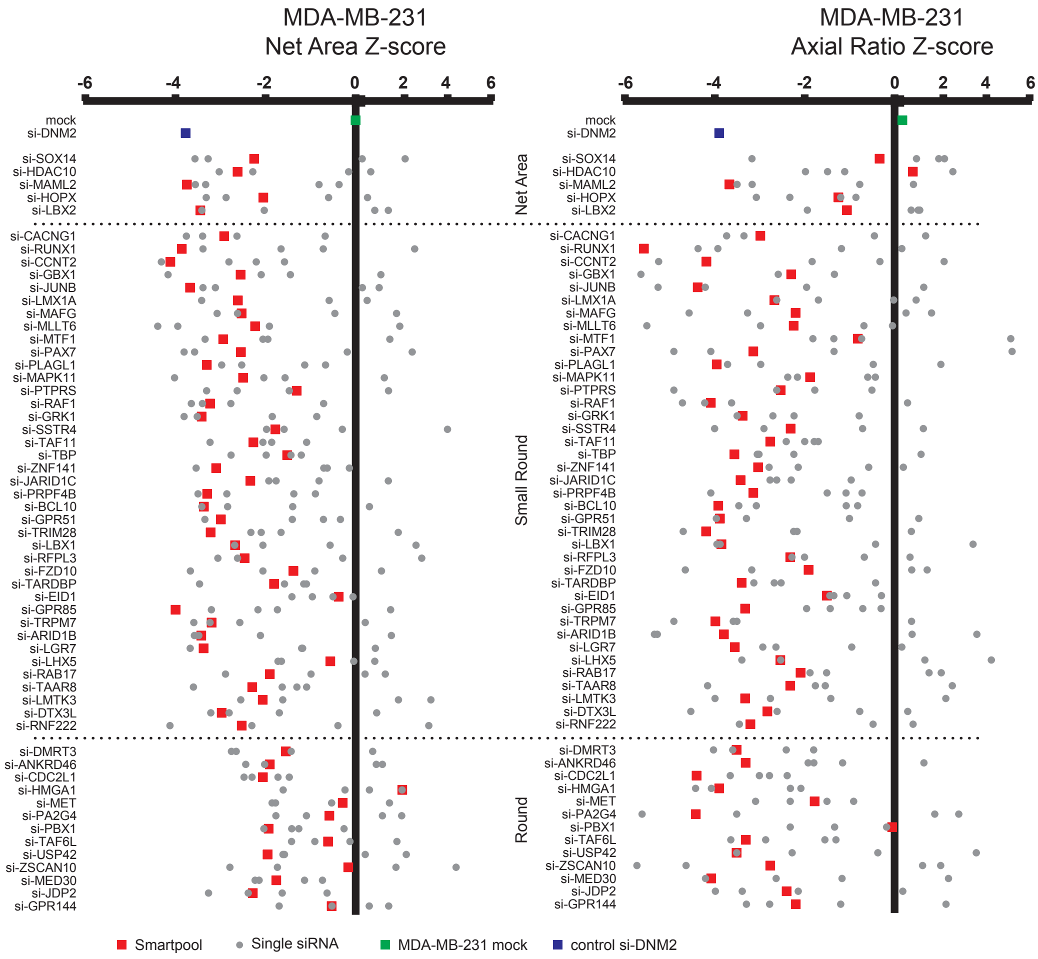

Supplementary Figure 4. ***Phenotypic candidate gene validation by deconvolution screens in MDA-MB-231.*** Hits were considered validated if SMARTpool and at least 2 of 4 single siRNAs showed the same effect. 282 selected genes were tested in a deconvolution PKT screen with 4 single siRNAs per gene. SMARTpool and single siRNA Z-scores of Net Area and Axial Ratio are shown for the 'overlap hits' that were validated in MDA-MB-231. Shown Z-scores are averages of technical and biological duplicates.

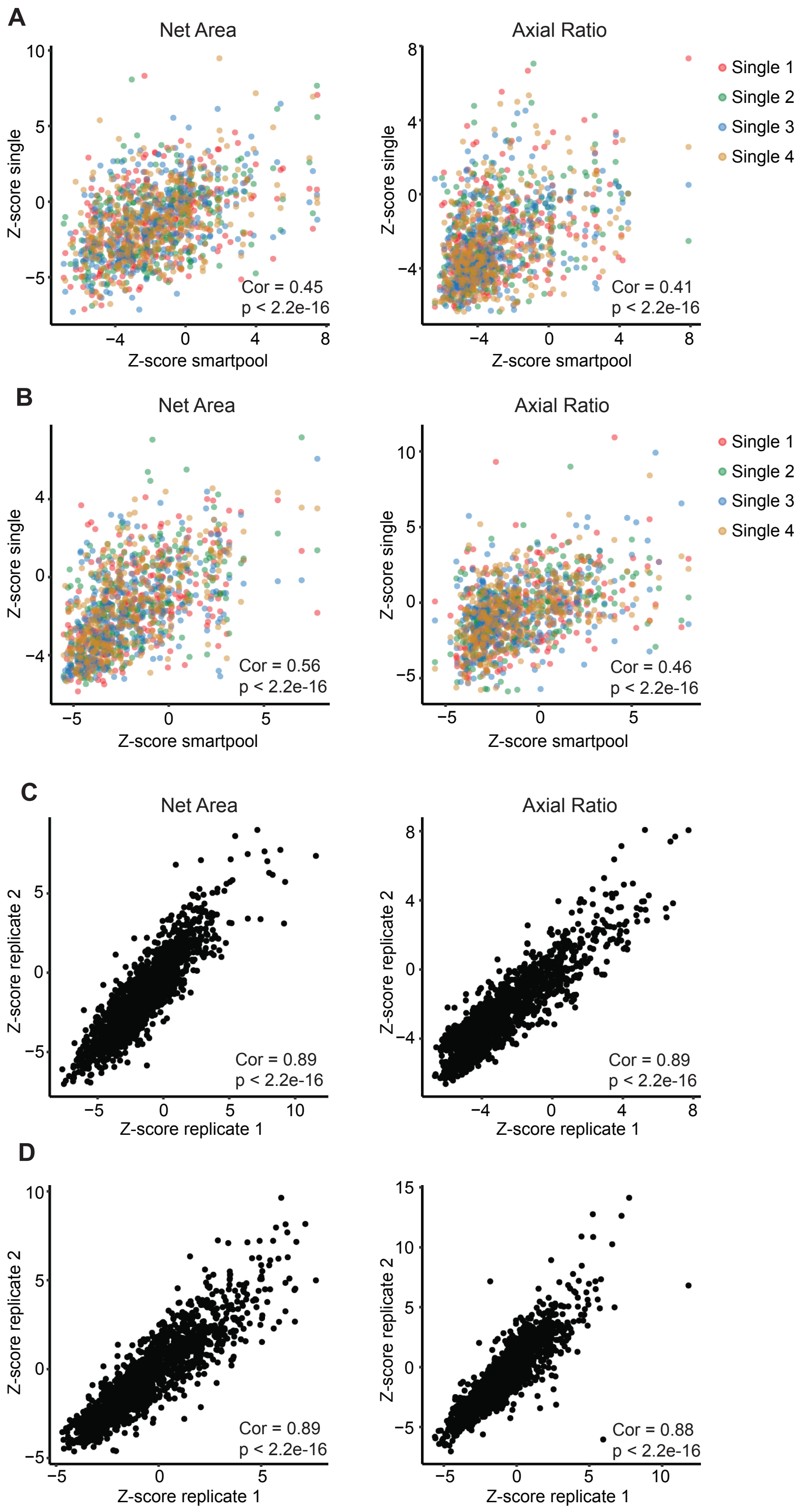

Supplementary Figure 5. ***Reproducibility of single siRNA PKT validation screen.*** (A) Correlation of Z-score of smartpool and single siRNAs in Hs578T. (B) Correlation of Z-score of smartpool and single siRNAs in MDA-MB-231. Shown Z-scores are averages of technical and biological duplicates. (C) Correlation of biological replicates in Hs578T. (D) Correlation of biological replicates in MDA-MB-231.

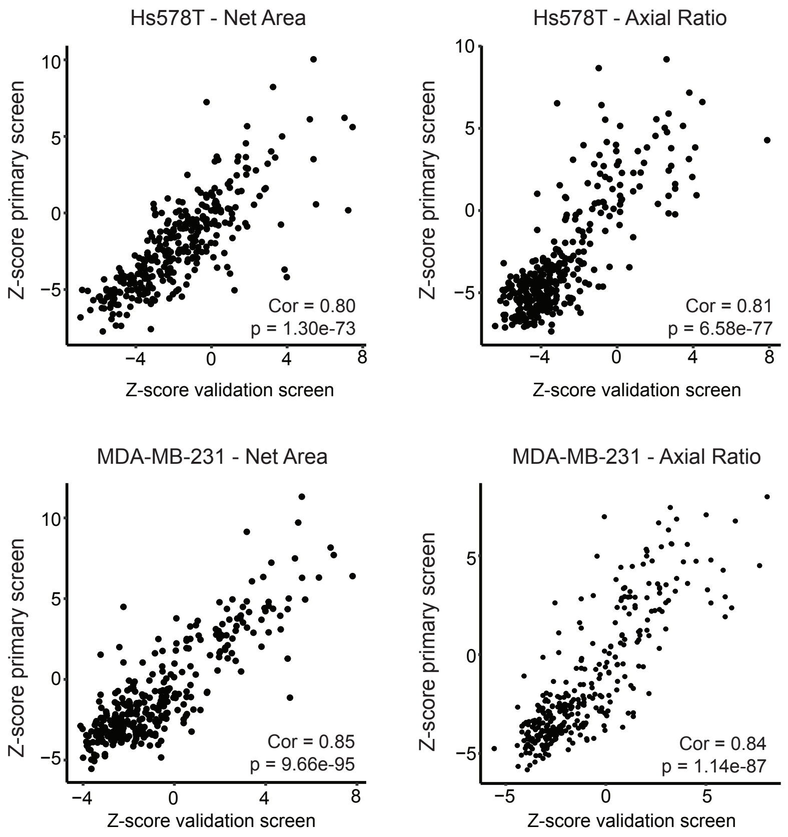

Supplementary Figure 6. ***Pearson correlation of Z-scores from primary screen and validation screen for net area and axial ratio track parameters in both Hs578T and MDA-MB-231.*** Shown Z-scores are averages of technical and biological duplicates.

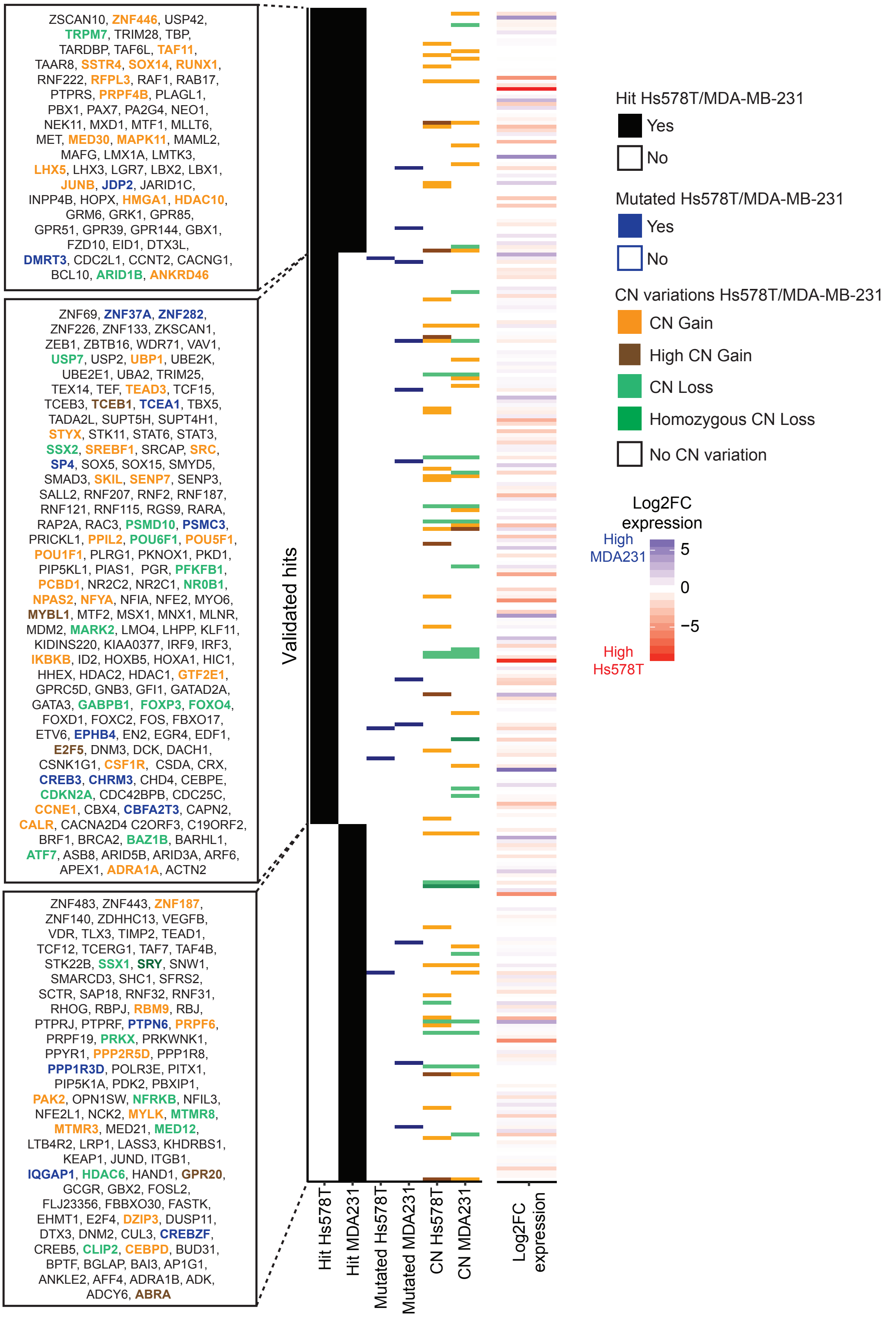

Supplementary Figure 7. ***Validated hits in Hs578T and MDA-MB-231 and cell line specific mutations, copy number alterations and differences in RNA expression levels.*** Candidates are in shown in cluster order, color indicates their mutation or copy number states in at least one of the cell lines (blue = mutated, yellow = copy number gain, brown = high copy number gain, light green = copy number loss, dark green = homozygous copy number loss).

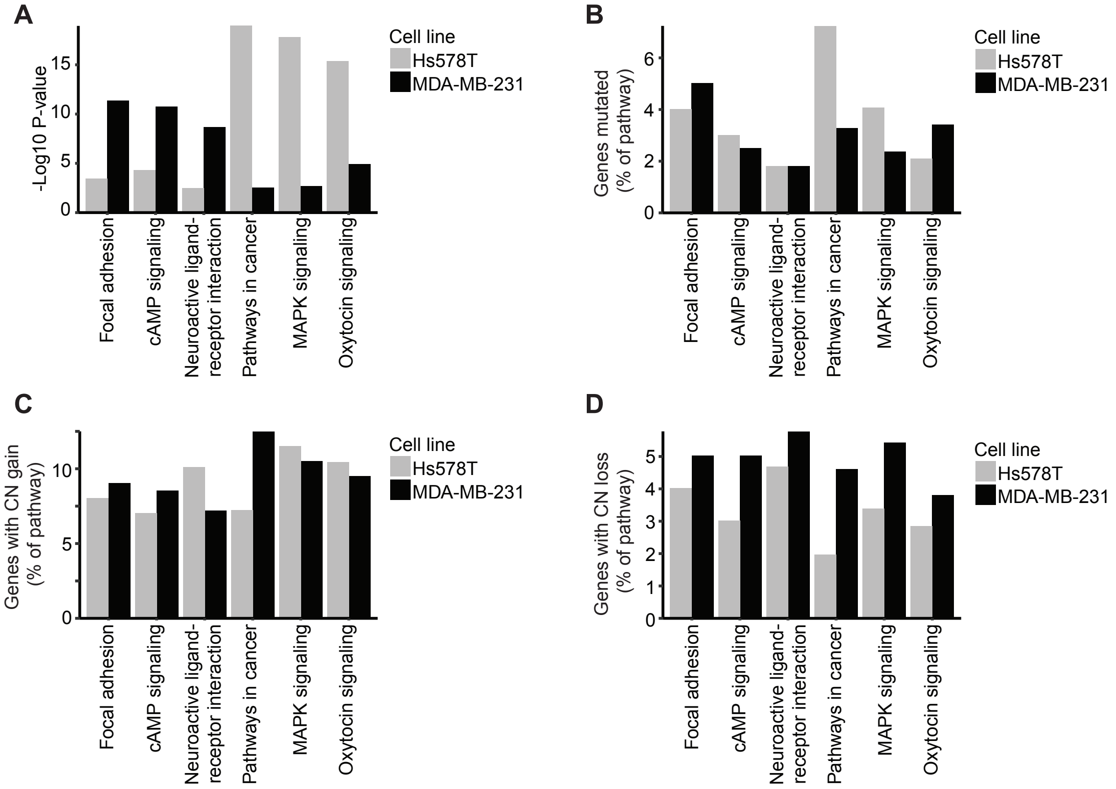

Supplementary Figure 8. ***Mutations and copy number alterations of over-represented pathways in Hs578T and MDA-MB-231 specific candidates.*** (A) Top pathways over-represented in primary screen candi-dates specific for MDA-MB-231 (left) or Hs578T (right) cell lines. Overrepresentation analysis was performed using all primary screen cell line hits using ConsensusPathDB. (B) Percentage of genes of selected pathways mutated in Hs578T and MDA-MB-231 cells. (C) Percentage of genes of selected pathways bearing copy number (CN) gain in Hs578T or MDA-MB-231 cells. (D) Percentage of genes of selected pathways bearing CN loss in Hs578T or MDA-MB-231 cells.

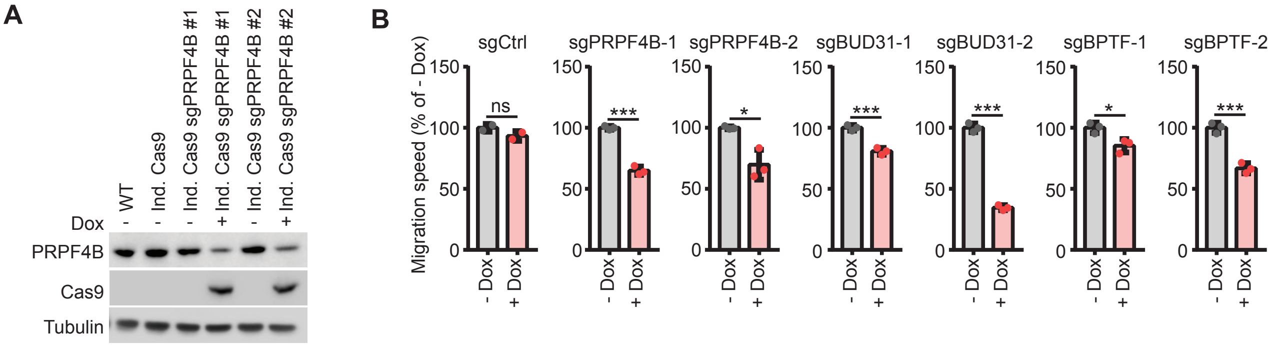

Supplementary Figure 9. ***Candidate CRISPR-Cas9 knockout and effect on live cell migration.*** (A) PRPF4B knockout efficiency 48 hours after Cas9 induction. (B) Migration speed of MDA-MB-231 ind-Cas9 72 hours after doxycyline exposure. The experiment was performed in biological triplicates except for sgCtrl (performed in biological duplicate), significance was calculated using student's t-test. * $p < 0.05$, ** $p < 0.01$, *** $p < 0.001$. Source data are provided as a Source Data file.

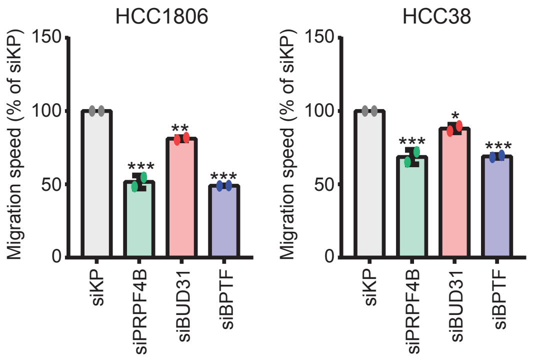

Supplementary Figure 10. ***Effect of PRPF4B, BUD31 and BPTF knockdown on live cell migration in HCC1806 and HCC38.*** Mean + stdev of two biological replicates. ANOVA with multiple testing correction was used for statistical analysis. * $p < 0.05$, ** $p < 0.01$, *** $p < 0.001$. Source data are provided as a Source Data file.

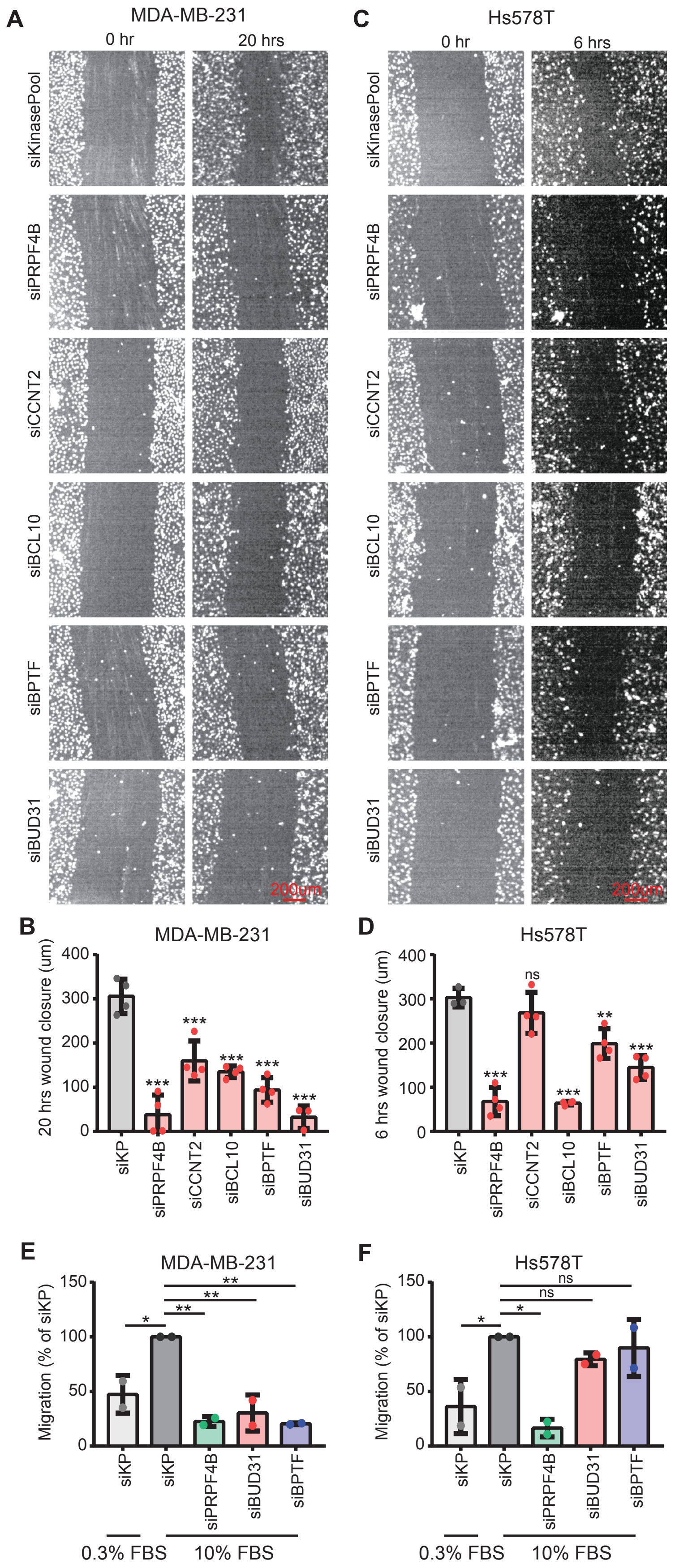

Supplementary Figure 11. ***Candidate knockdown effects on cell migration in scratch and Boyden Cham-ber assays.*** (A) Representative images of the wound size 0 and 20 hours after scratch preparation in MDA-MB-231 72 hours after candidate knockdown. (B) Quantification of A. Bars represent mean + sd, n = 4. (C) Representative images of wound size 0 and 6 hours after scratch preparation in Hs578T 72 hours after candidate knockdown. (D) Quantification of A. Bars represent mean+sd, n = 4. (E) Directed cell migration to FBS of candidate knockdowns (65 hours after transfection) in MDA-MB-231 using a Boyden Chamber assay. Migration is normalized to siKP knockdown. Bars show mean + sd of 2 biological replicates. (F) Same as in E, but for Hs578T. Significance was determined using ANOVA correcting for multiple testing. * p < 0.05, ** p < 0.01, *** p < 0.001. Source data are provided as a Source Data file.

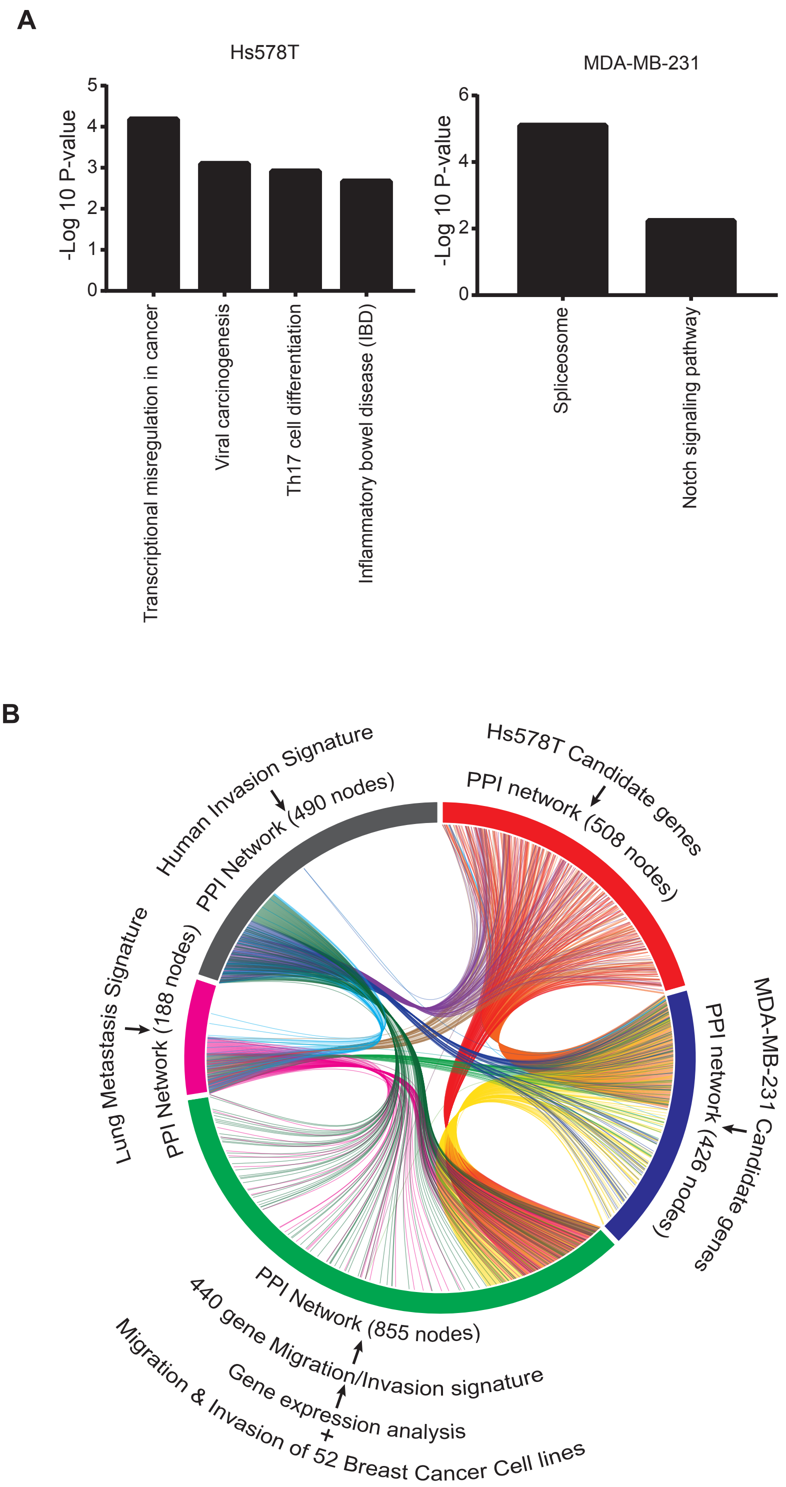

Supplementary Figure 12. ***Interaction between candidate genes and migration signatures.*** (A) Enrichment of KEGG Pathways in Hs578T and MDA-MB-231 validated candidate genes. (B) Chord diagram displaying the connection between our cell migration networks and PPI networks derived from cell migration signatures.

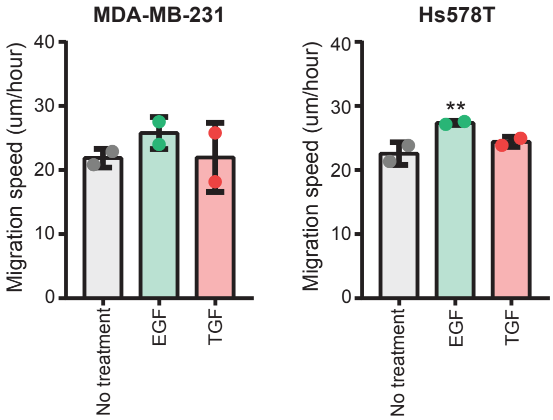

Supplementary Figure 13. ***Effect of TGF-beta treatment on TNBC cell migration.*** Random cell migration speed of MDA-MB-231 or Hs578T cells after 2 hours starvation, followed by EGF (100 ng/ml) or TGF (10 ng/ml) treatment. Average and standard deviation of two independent replicates are shown. Significance was deter-mined using ANOVA correcting for multiple testing. * $p < 0.05$. Source data are provided as a Source Data file.

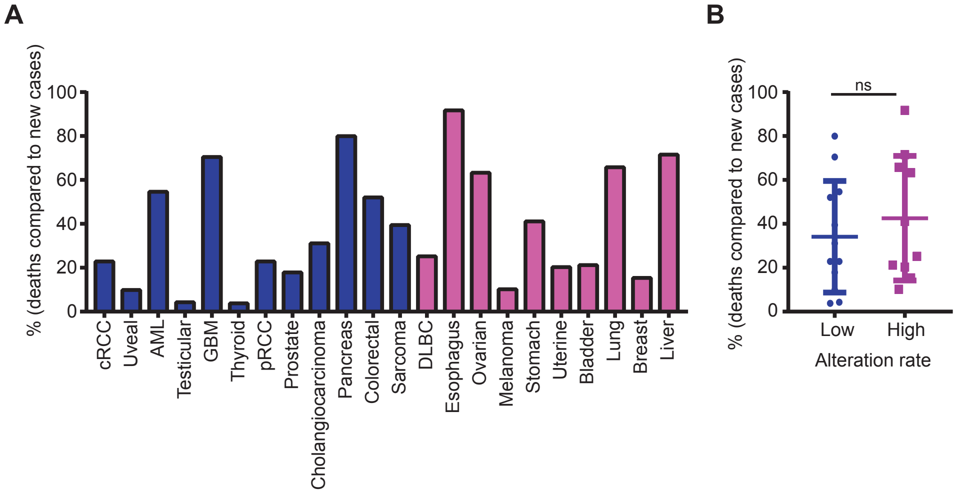

Supplementary Figure 14. ***Tumor subtype aggressiveness.*** (A) % of deaths compared to new cases for different cancer types estimated for 2018 in the United States. Color indicates the alteration rate for the different candidates. Blue = low alteration rate, pink = high alteration rate. (B) Same as A, now ordered by alteration rate.

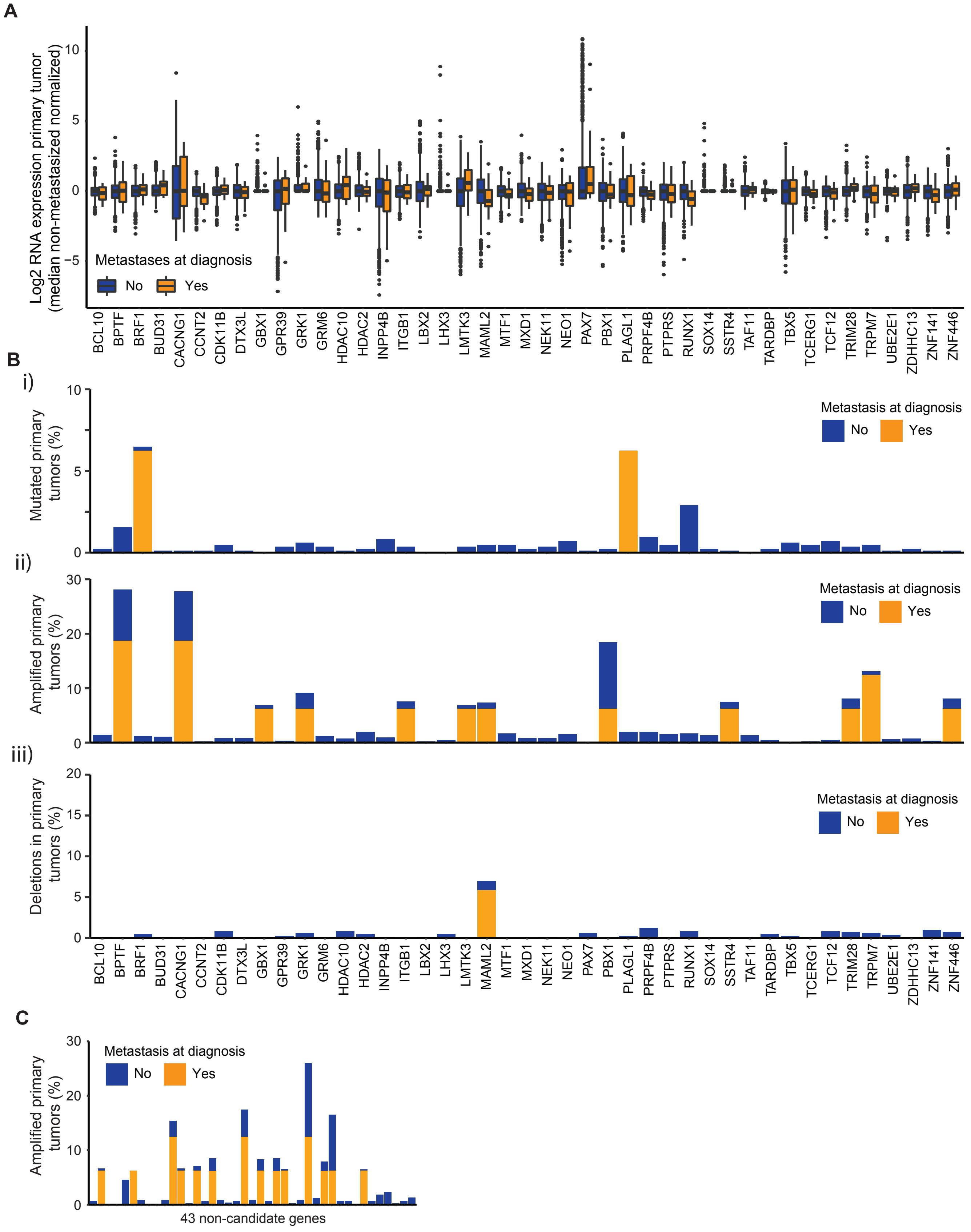

Supplementary Figure 15. ***Candidate gene alterations in primary tumors with and without metastasis at diagnosis.*** (A) Log2 RNA expression levels of candidates in primary tumors with (orange, n=22) or without (blue, n=906) distant metastases at diagnosis. Boxplots represent median, first and third quartile. (B) Candidate gene mutation rate (i), amplification rate (ii) and deletion rate (iii) in primary tumors with (orange, n=22) or with-out (blue, n=906) metastases at diagnosis. (C) Amplification of randomly selected non-hits in primary tumors with and without metastasis at diagnosis. Significance was calculated using the Fisher's exact test using the Benjamini-Hochberg method to correct for multiple testing. * $p < 0.05$, ** $p < 0.01$, *** $p < 0.001$.

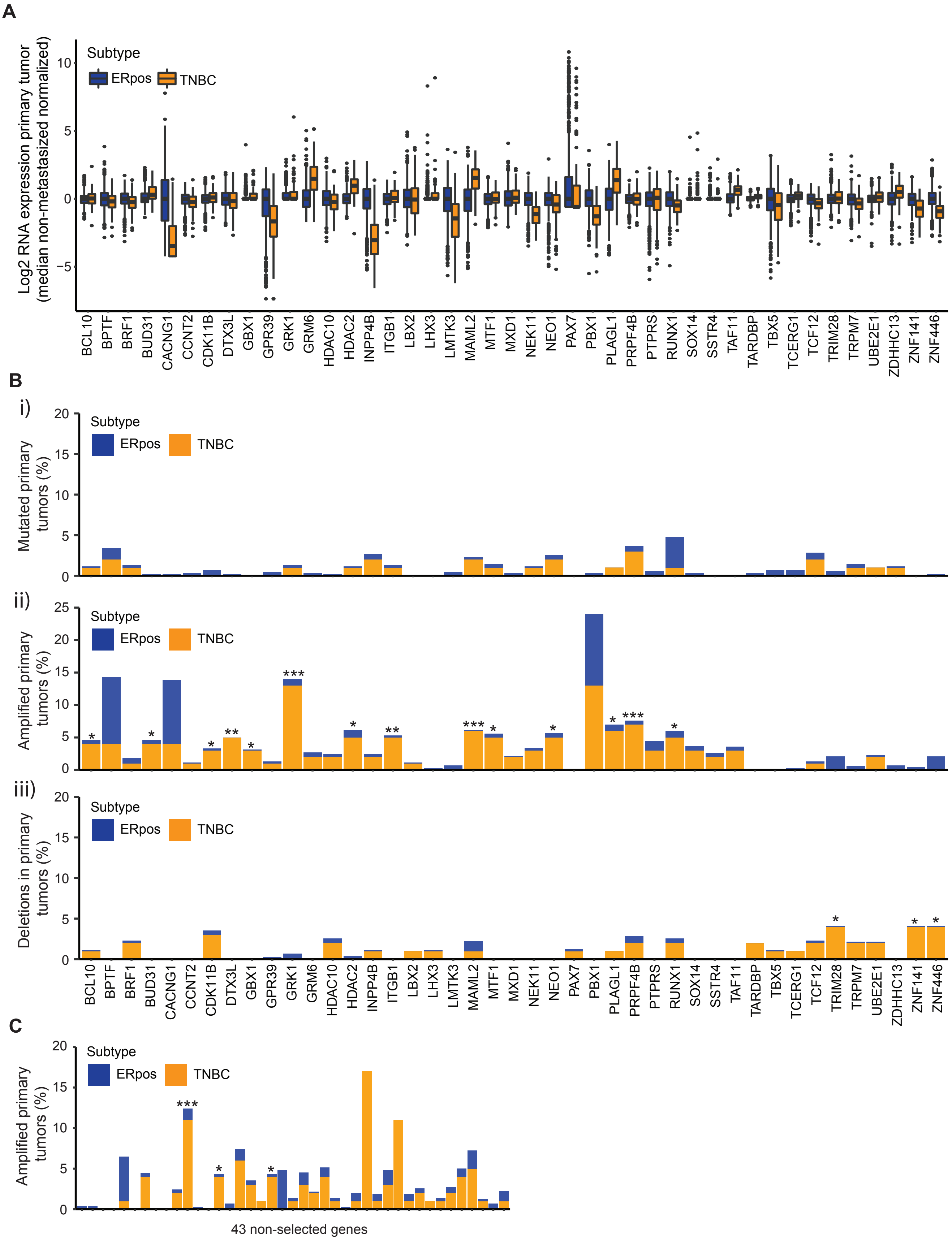

Supplementary Figure 16. ***Candidate gene alterations in ERpos and TNBC primary breast tumors.*** (A) Log2 RNA expression levels of candidates in ERpos (n=808) and TNBC primary tumors (n=116). Boxplots represent median, first and third quartiles. (B) Candidate gene mutation rate (i), amplification rate (ii) and dele-tion rate (iii) in ERpos and TNBC primary tumors. (C) Amplifcation of randomly selected non-hits in ERpos (n=808) and TNBC (n=116) primary tumors. Significance was calculated using the Fisher's exact test using the Benjamini-Hochberg method to correct for multiple testing. * $p < 0.05$, ** $p < 0.01$, *** $p < 0.001$.

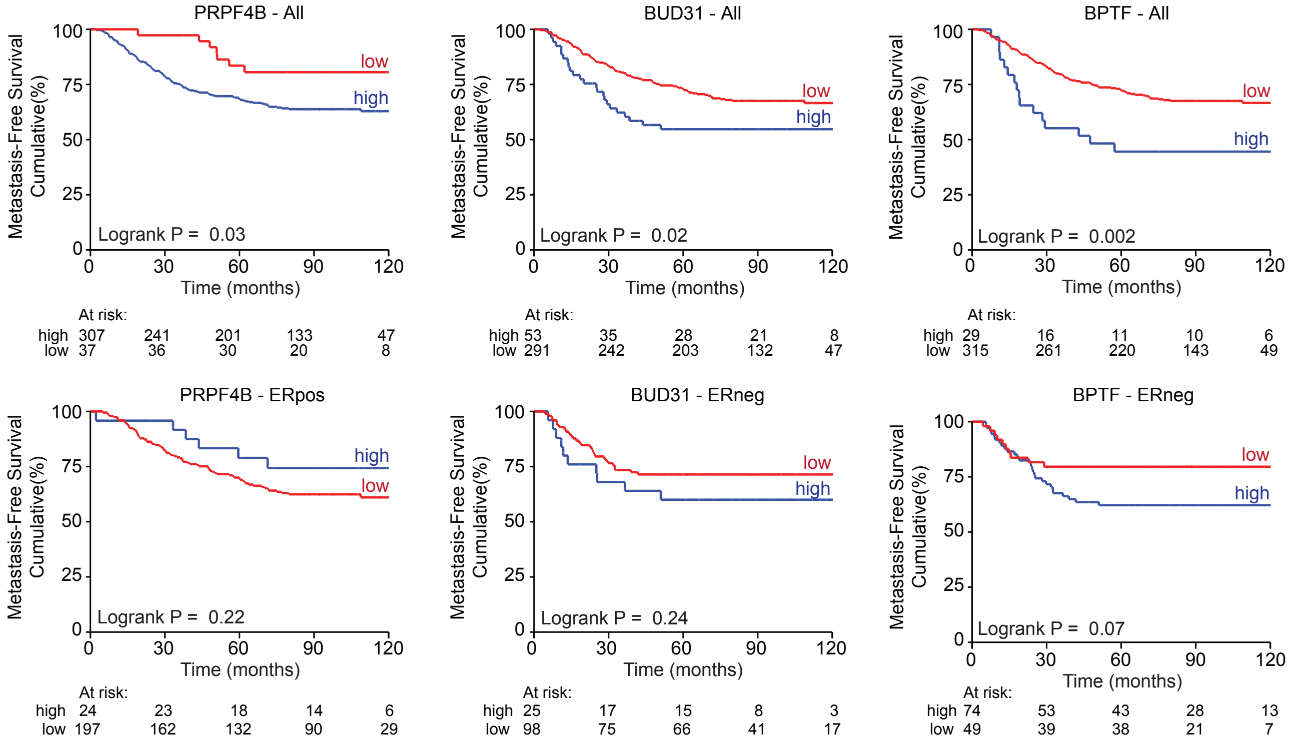

Supplementary Figure 17. ***Candidate modulators of TNBC cell migration are related to distant metasta-sis-free survival in breast cancer patients.*** Kaplan Meier curves for expression of PRPF4B, BUD31 and BPTF and relation to metastasis-free survival in All, ERpos or ERneg breast cancer patients. Gene expression data of lymph-node negative BC patient cohort without prior treatment using optimal split was used to obtain the curves.

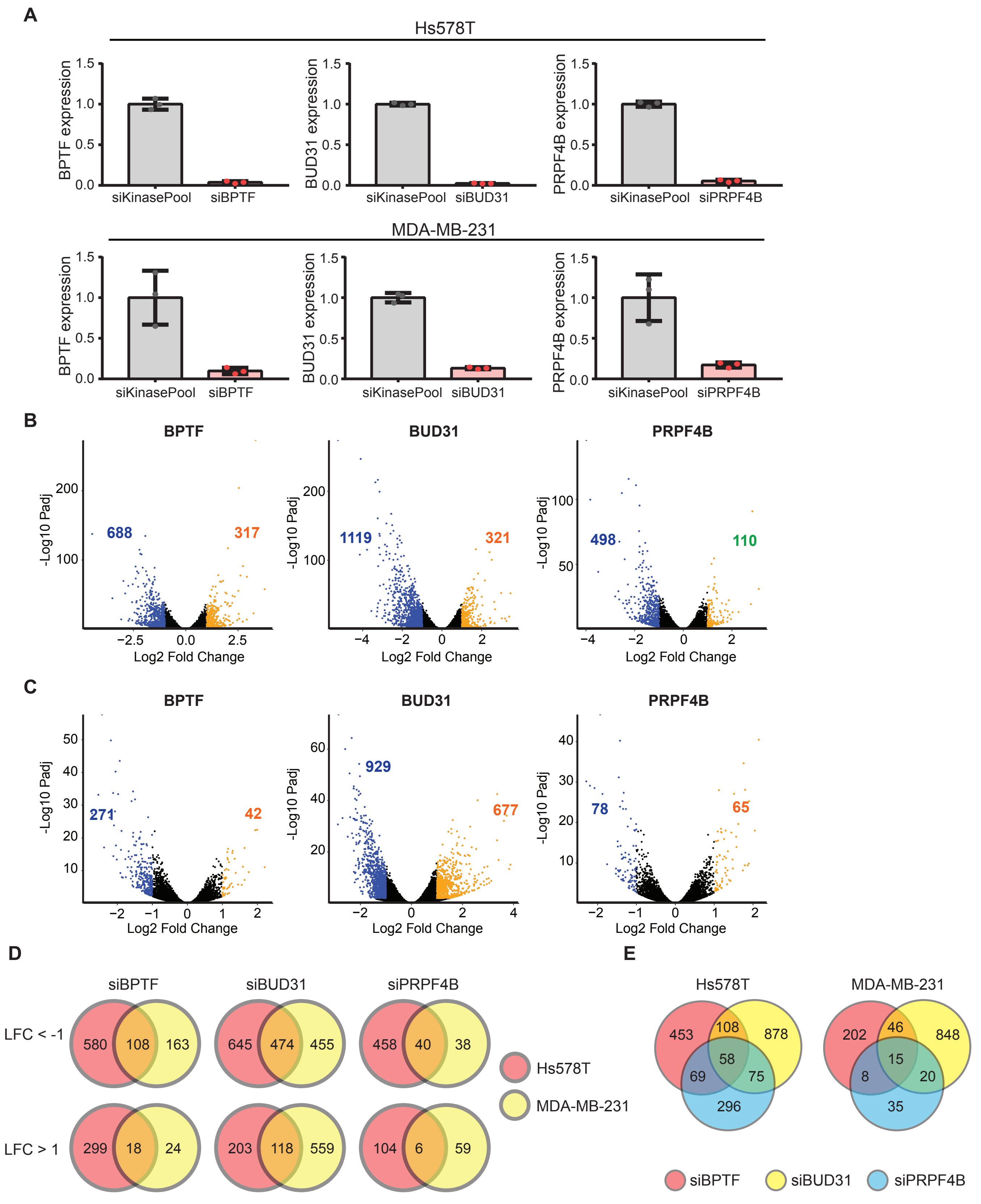

Supplementary Figure 18. *Effect of PRBF4B, BUD31 and BPTF depletion on gene expression.* (A) qRT-PCR of knockdown efficiency of siPRBF4B, siBUD31 and siBPTF used for next generation sequencing in Hs578T and MDA-MB-231 cells. Data are normalized using the ΔΔCT method normalized to actin and tubulin levels. Mean + sd of three biological replicates. Significance was determined using a Student's T-test. Source data are provided as a Source Data file. (B) Vulcano plots for Hs578T. Significant up- and down-regulated genes (DEGs, L2FC > 1 or < -1 and P-adjusted < 0.01) are shown in orange and blue, respectively. L2FC com-pared to siKP, mean of 3 biological replicates, significance is determined using the DESeq2 R package (for further details, see the methods section). (C) Same as in B for MDA-MB-231. (D) Overlap of DEGs in Hs578T and MDA-MB-231. (E) Overlap of DEGs comparing different knockdown conditions.

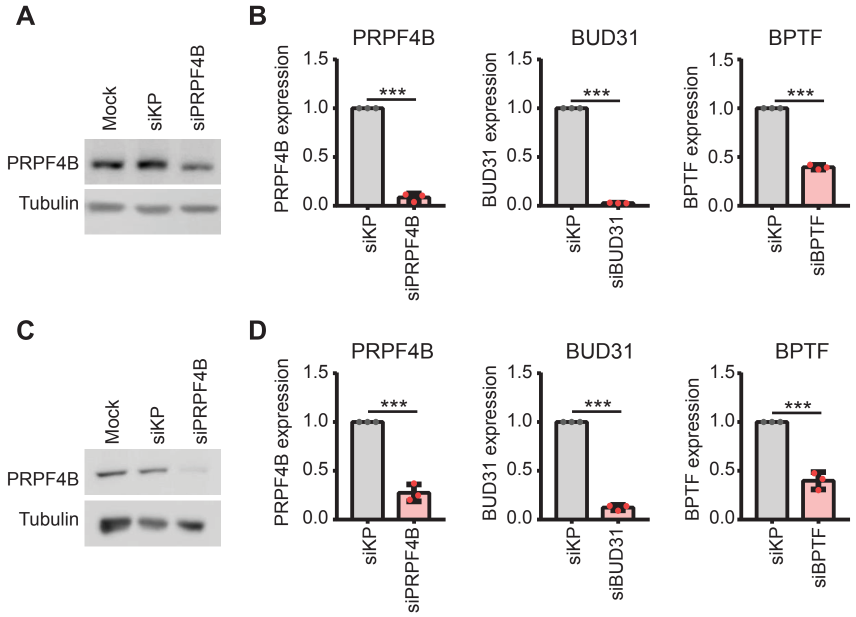

Supplementary Figure 19. ***Candidate siRNA knockdown efficiency in Hs578T and MDA-MB-231.*** (A) PRPF4B knockdown efficiency on protein level in Hs578T. (B) PRPF4B, BUD31 and BPTF knockdown efficien-cy on RNA level in Hs578T. (C) PRPF4B knockdown efficiency on protein level in MDA-MB-231. (D) PRPF4B, BUD31 and BPTF knockdown efficiency on RNA level in MDA-MB-231.Experiments were performed in biologi-cal triplicates, significance was calculated using student's t-test. *** $p < 0.001$. Source data are provided as a Source Data file.

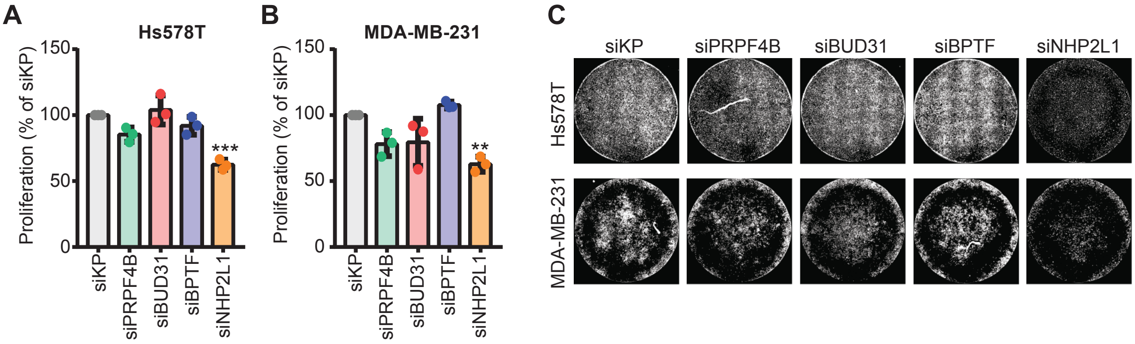

Supplementary Figure 20. ***Effect of PRPF4B, BUD31 and BPTF knockdown on cell proliferation.*** Prolifera-tion of candidate knockdown compared to siKinasePool knockdown in (A) Hs578T and (B) MDA-MB-231 cells 72 hours after knockdown using the SRB assay. Mean + stdev of three biological replicates. Significance was determined using one-way ANOVA, using correction for multiple hypothesis testing. ** p<0.01, *** p<0.001. (C) Representative 96-well images of nuclei staining in Hs578T and MDA-MB-231 cells with candidate or control knockdown. Source data are provided as a Source Data file.

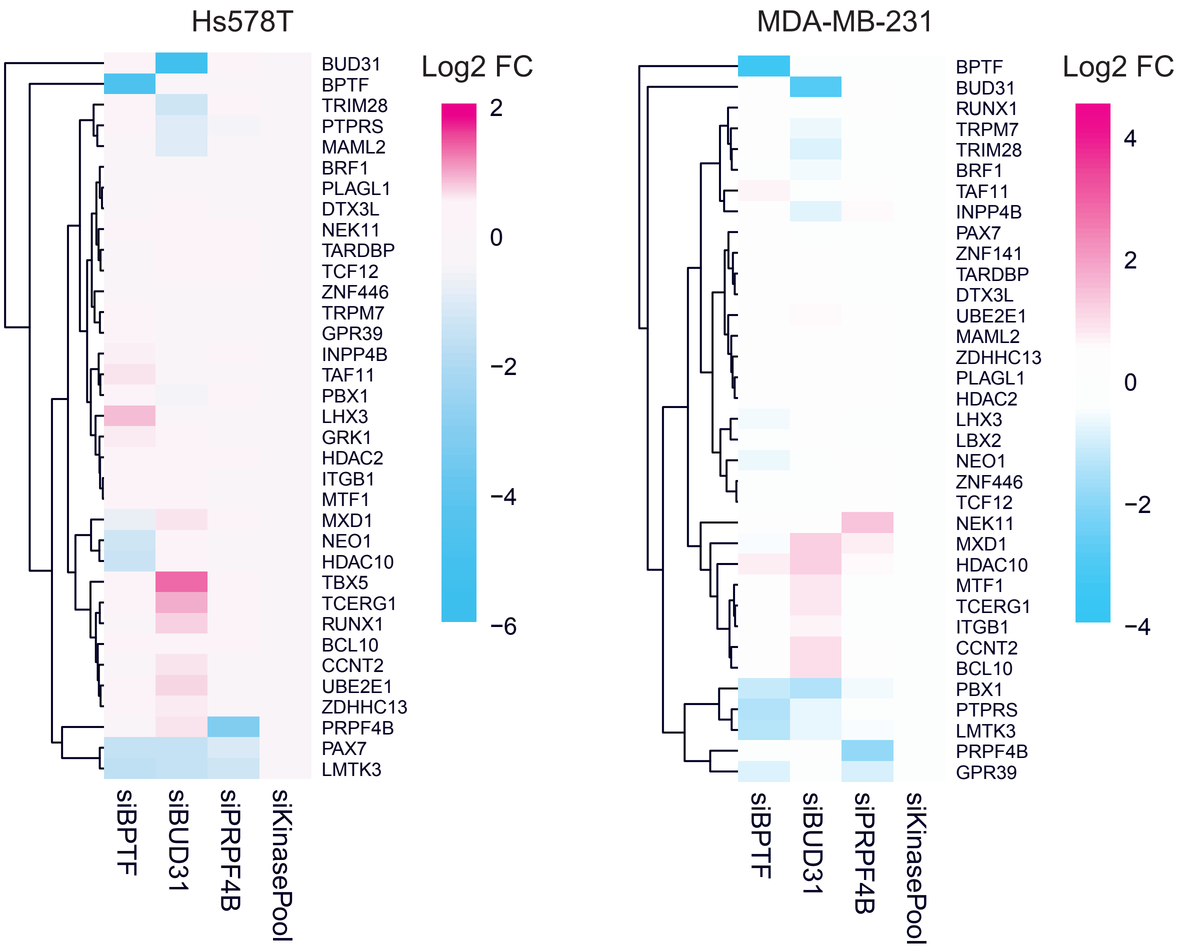

Supplementary Figure 21. ***Effect of PRBF4B, BUD31 and BPTF depletion on expression of other candi-date TBNC cell migration modulators.*** Hierarchical clustering (Euclidean distance, complete linkage) of log2FC of the gene expression of 35 common validated candidate TNBC cell migration modulators after knock-down of BUD31, PRPF4B or BPTF in Hs578T and MDA-MB-231. The shown log2FC is the average of 3 biologi-cal replicates.

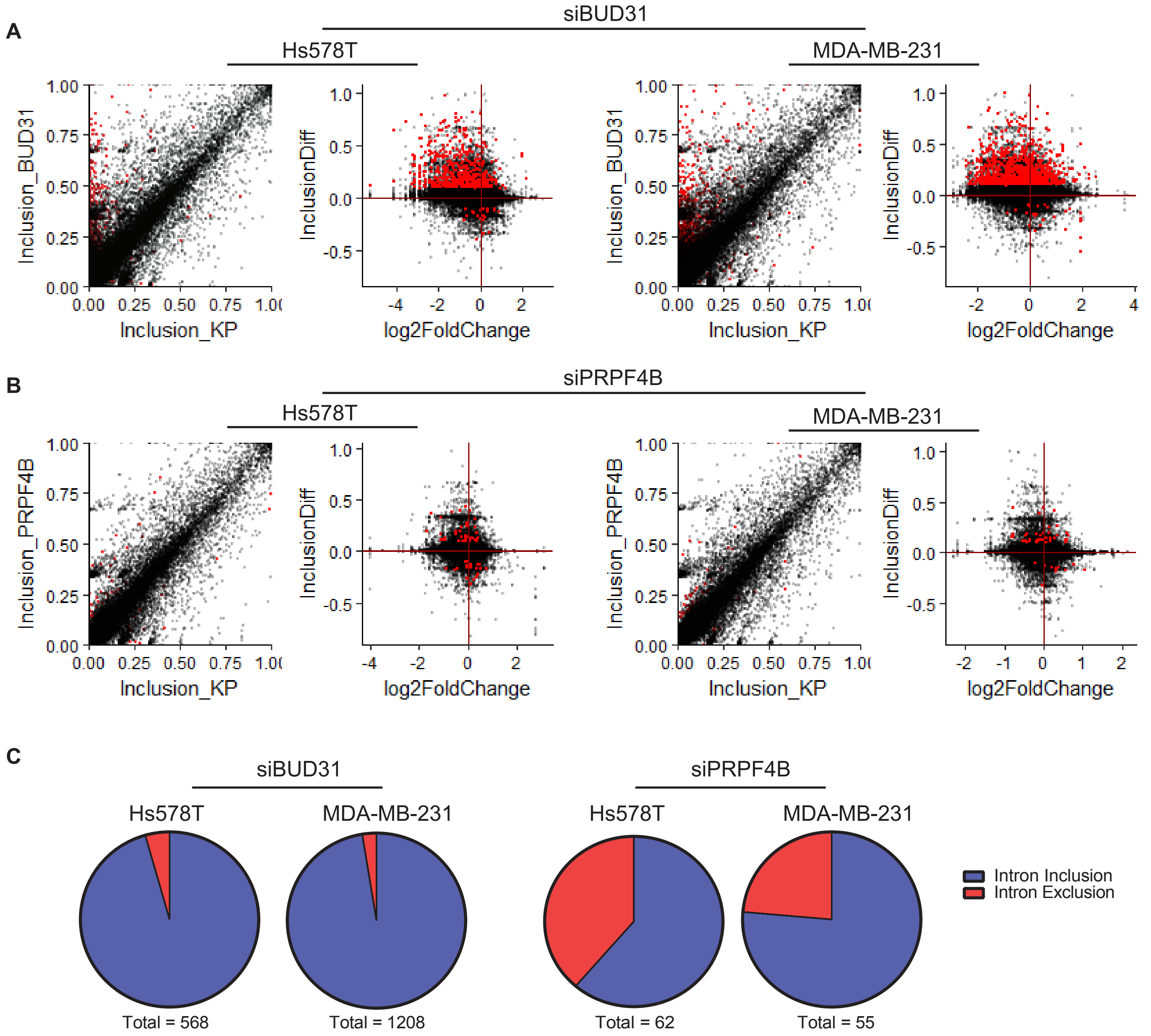

Supplementary Figure 22. ***Effect of BUD31 and PRPF4B depletion on intron retention and exclusion.*** (A) Effect of siBUD31 on intron retention. For all introns, the fraction of siBUD31 versus siKP is plotted. Introns for which the inclusion rate significantly differed from siKP are plotted in red (inclusion difference > 0.10 and padj < 0.01). The inclusion difference plotted against the log2FC in expression level demonstrates that intron inclu-sion in general results in lower expression of these genes. (B) The same as in A, but then for siPRPF4B knock-down. (C) The total number of intron inclusion and exclusion events in siBUD31 and siPRPF4B samples. All shown values are the average of three biological replicates.

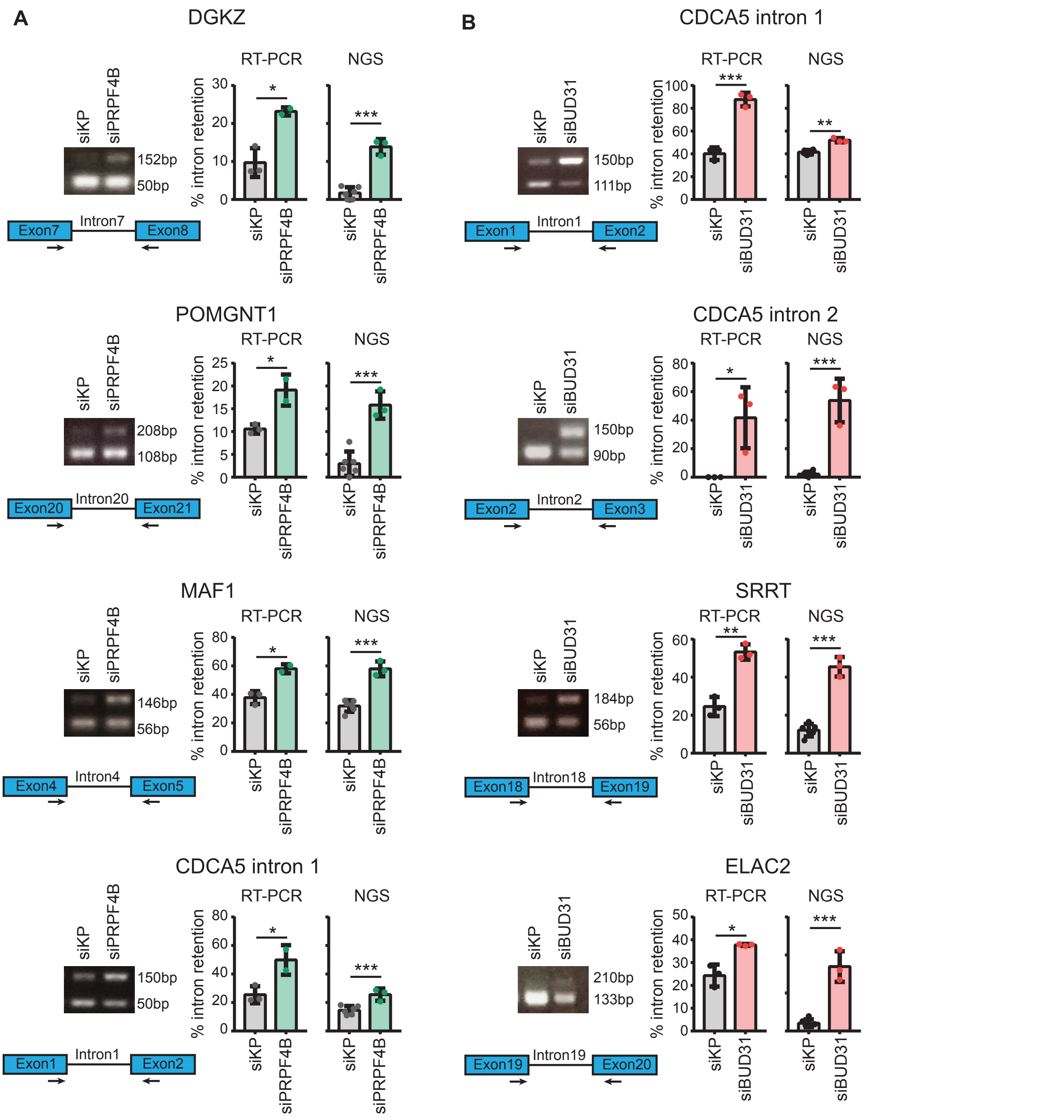

Supplementary Figure 23. ***Validation of intron retention events in MDA-MB-231.*** (A) RT-PCR validation of PRPF4B knockdown induced alternative splicing events in MDA-MB-231 detected by NGS 72 hours after trans-fection. (B) RT-PCR validation of BUD31 knockdown induced alternative splicing events in MDA-MB-231 detected by NGS 72 hours after transfection. Mean + stdev of three biological replicates. Significance is deter-mined using a student's t-test.* $p < 0.05$, ** $p < 0.01$, *** $p < 0.001$. Source data are provided as a Source Data file.

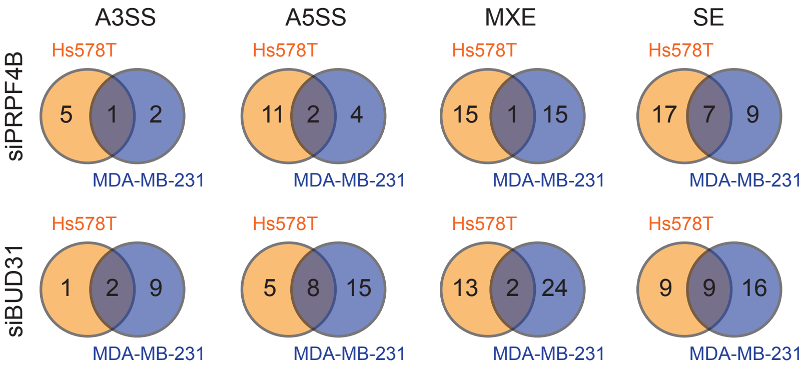

Supplementary Figure 24. ***Overlap of genes alternatively spliced between different cell lines.*** A3SS = alternative 3' splice site usage. A5SS = alternative 5' splice site usage. MXE = mutually exclusive exon. SE = skipped exon.

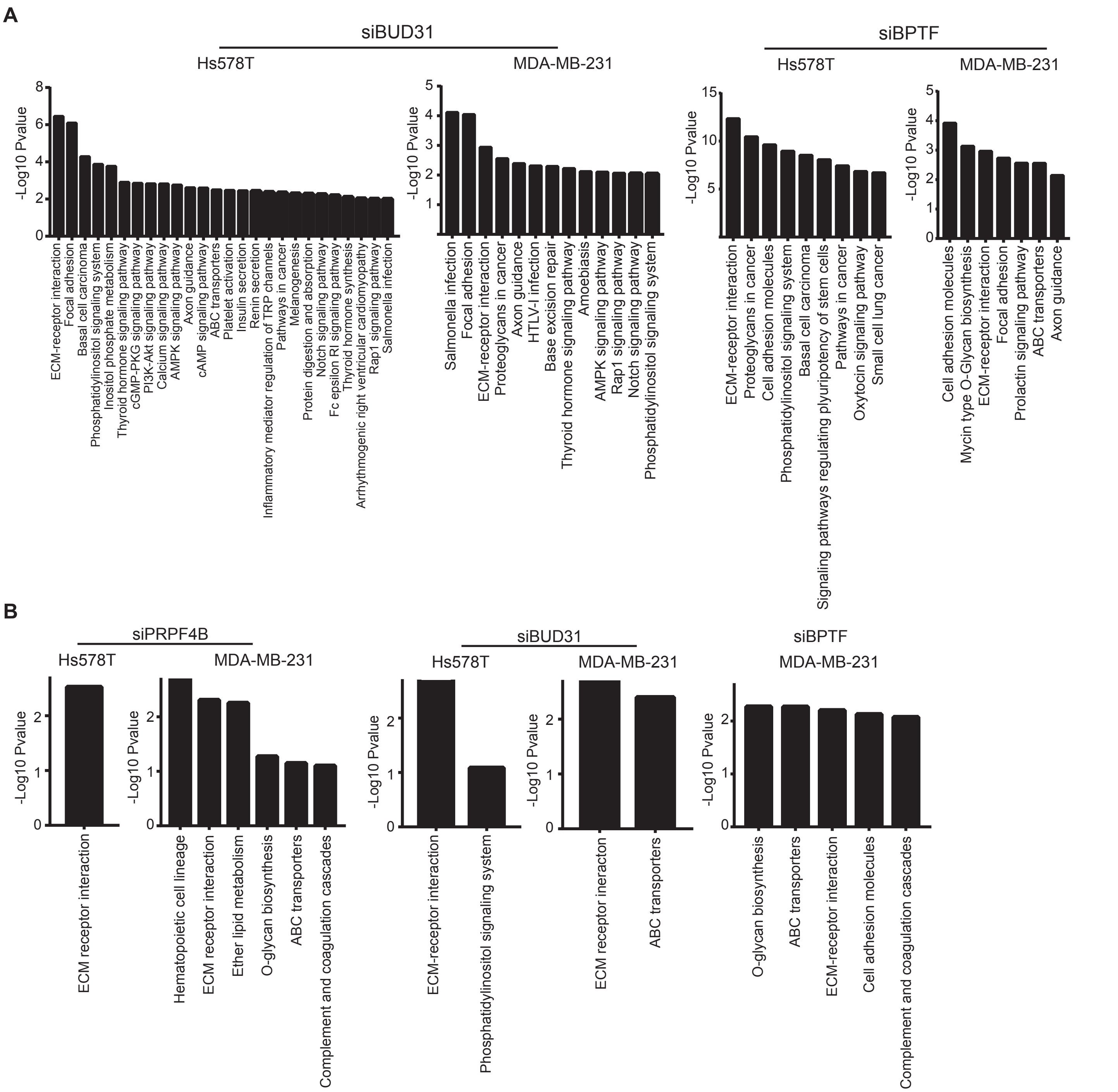

Supplementary Figure 25. ***Pathway analysis of altered gene expression after PRPF4B, BUD31 and BPTF depletion.*** (A) Significantly over-represented KEGG pathways in the downregulated DEGs (Log2FC < -1) after BUD31 or BPTF knockdown in the Hs578T or MDA-MB-231 cell line. (B) Significantly inhibited KEGG pathways after PRPF4B, BUD31 or BPTF knockdown in Hs578T and MDA-MB-231 cell line identified by performing a ranked gene set enrichment analysis (GSEA).

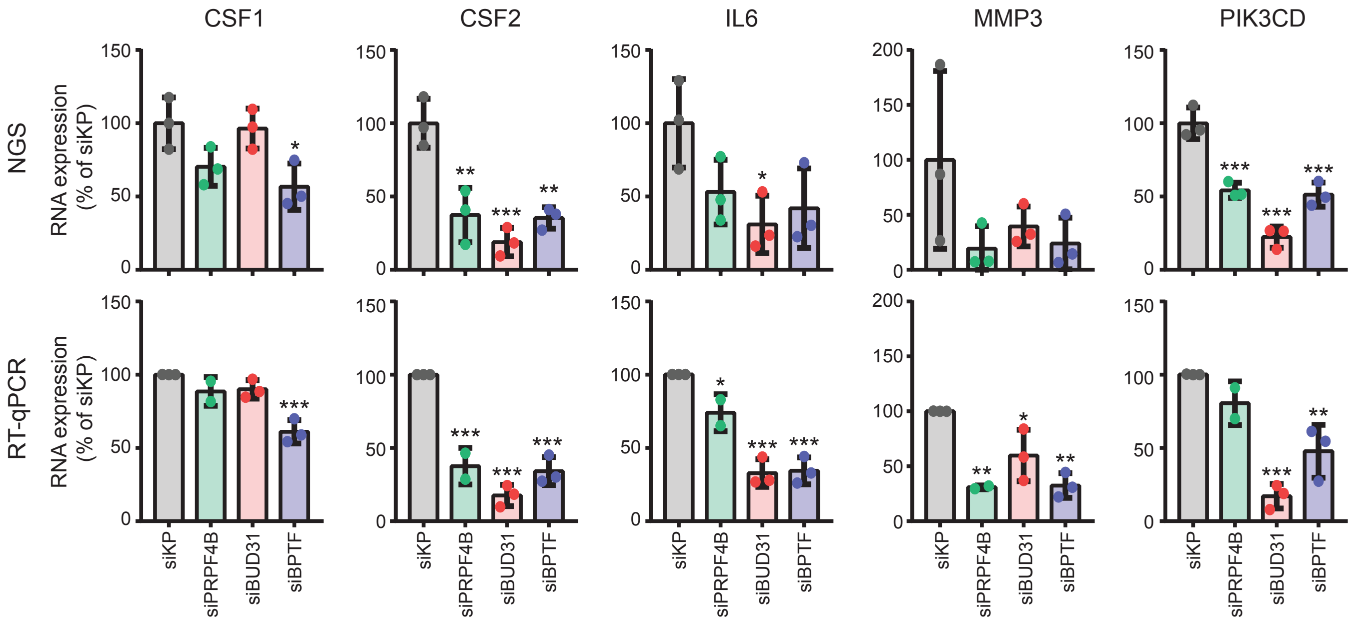

Supplementary Figure 26. ***Candidate knockdown results in deregulated TNF-alpha signaling.*** Effect of candidate knockdown on TNF-alpha signaling components CSF1, CSF2, IL6, MMP3 and PIK3CD in next gen-eration sequencing (top) and RT-qPCR (bottom) 72 hours after knockdown. Mean + stdev of 3 biologically inde-pendent replicates. Significance was determined using ANOVA correcting for multiple testing. * p < 0.05, ** p < 0.01, *** p < 0.001. Source data are provided as a Source Data file.

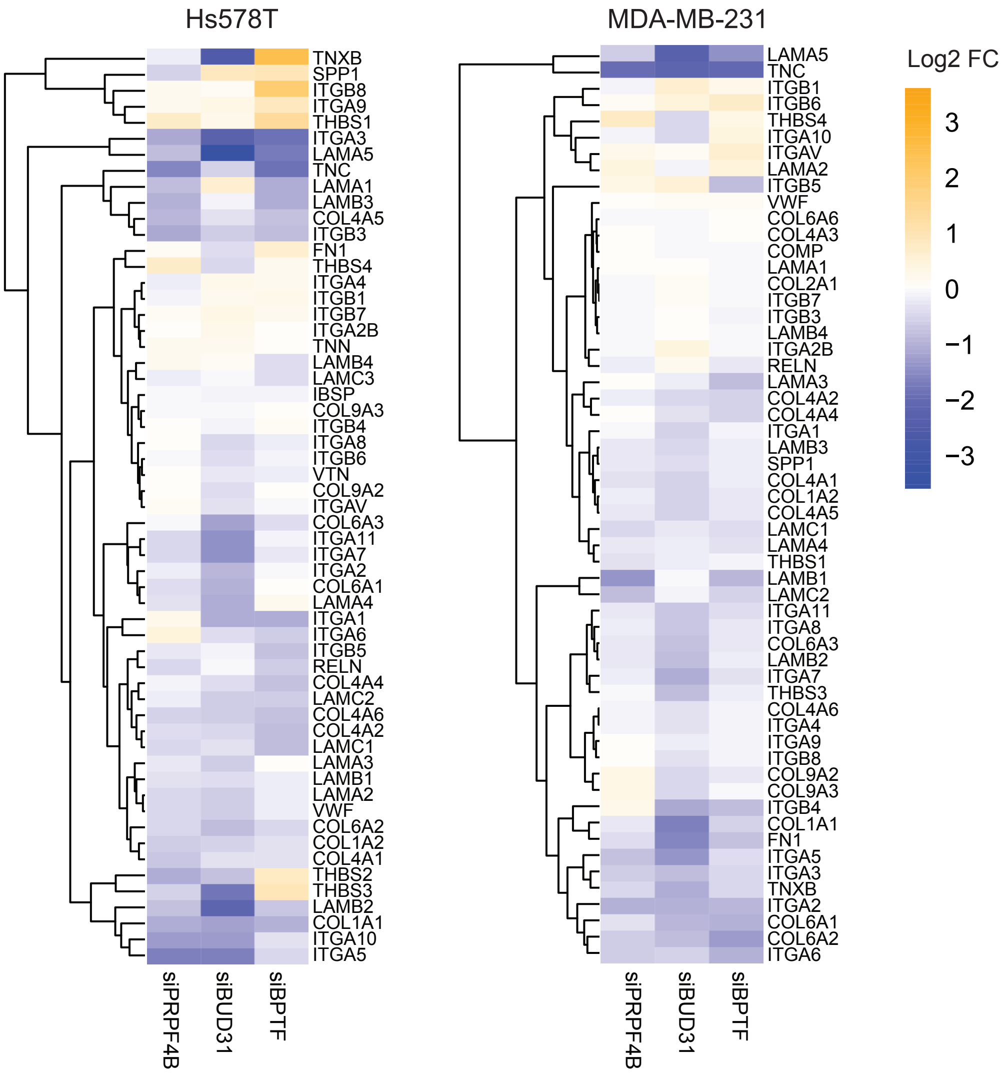

Supplementary Figure 27. ***Effect of PRPF4B, BUD31 or BPTF depletion on levels of ECM components.*** Hierarchical clustering (Euclidean distance, complete linkage) of log2FC in expression levels of genes involved in the KEGG ECM receptor interaction pathway in Hs578T and MDA-MB-231 72 hours after knockdown of PRPF4B, BUD31 or BPTF. The log2FC is the average of 3 biological replicates. Zoom in of Fig. 6C.

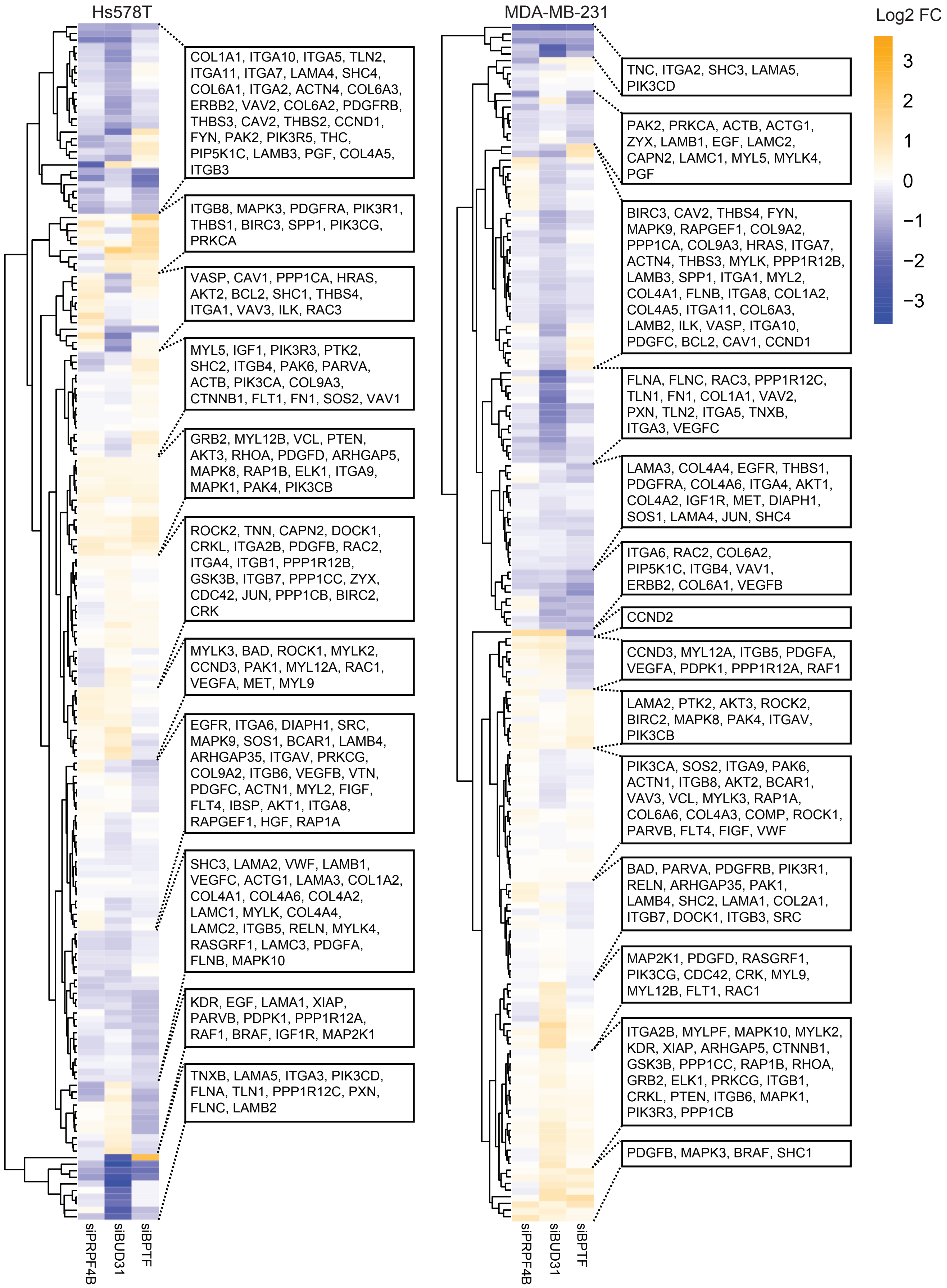

Supplementary Figure 28. ***Effect of PRPF4B, BUD31 or BPTF depletion of levels of focal adhesion com-ponents.*** Hierarchical clustering (Euclidean distance, complete linkage) of log2FC in expression levels of genes involved in the KEGG focal adhesion pathway in Hs578T and MDA-MB-231 72 hours after PRPF4B, BUD31 or BPTF knockdown. The log2FC is the average of 3 biological replicates.

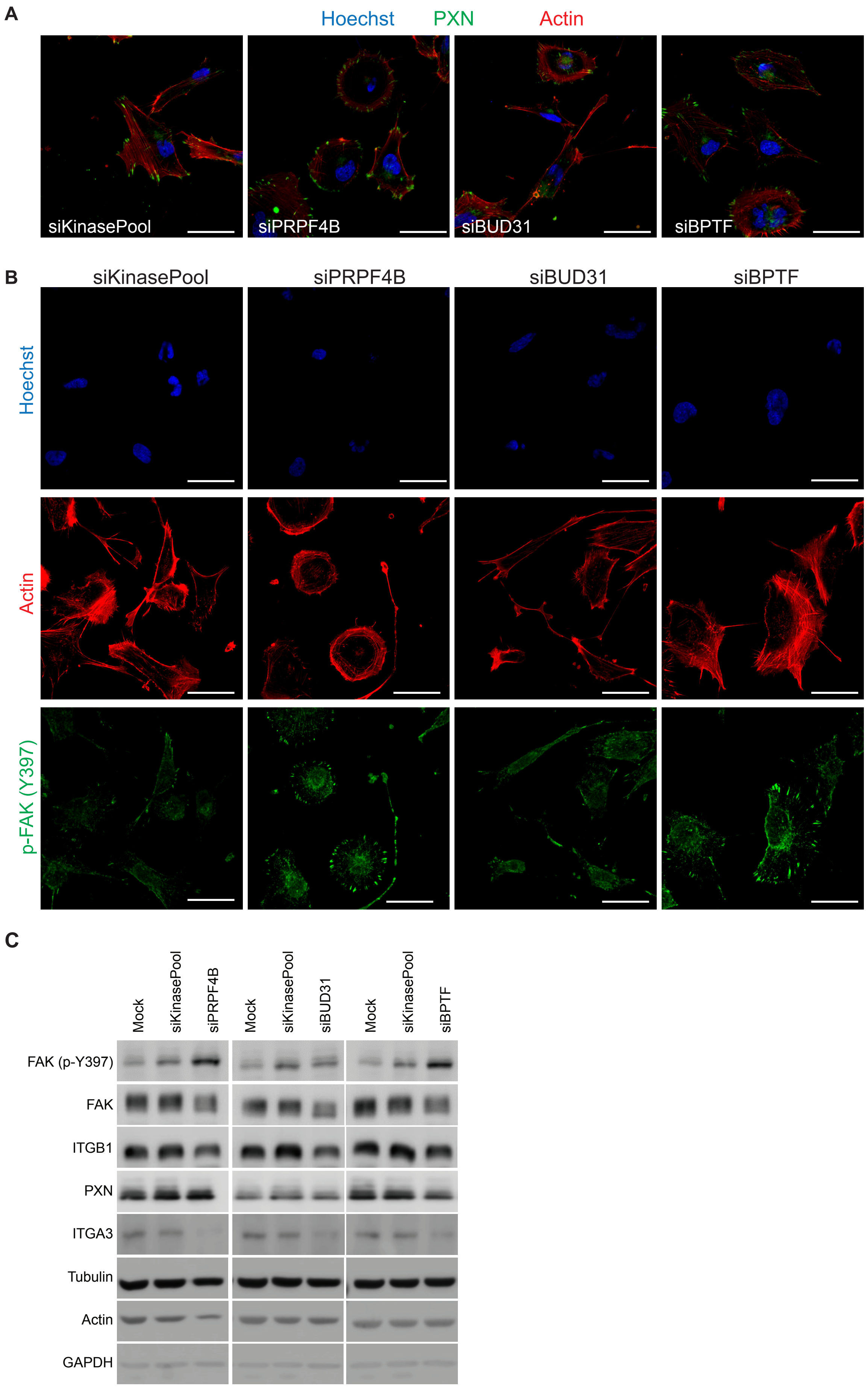

Supplementary Figure 29. ***Effect of PRPF4B, BUD31 or BPTF depletion on cell phenotype.*** (A) Effect of indicated gene depletion on focal adhesion and actin cytoskeleton organization three days after transfection. Hs578T cells were fixed and stained against the actin cytoskeleton, paxillin (PXN) and Hoechst (nuclei stain-ing). (B) Hs578T cells were fixed and stained against the actin cytoskeleton, p-FAK(Y397) and Hoechst as in Figure 6F. Scale bar is 50 µm. (C) Protein levels of focal adhesion and ECM-interaction related components after 72 hours after knockdown of PRPF4B, BUD31 or BPTF of candidates in Hs578T cells.

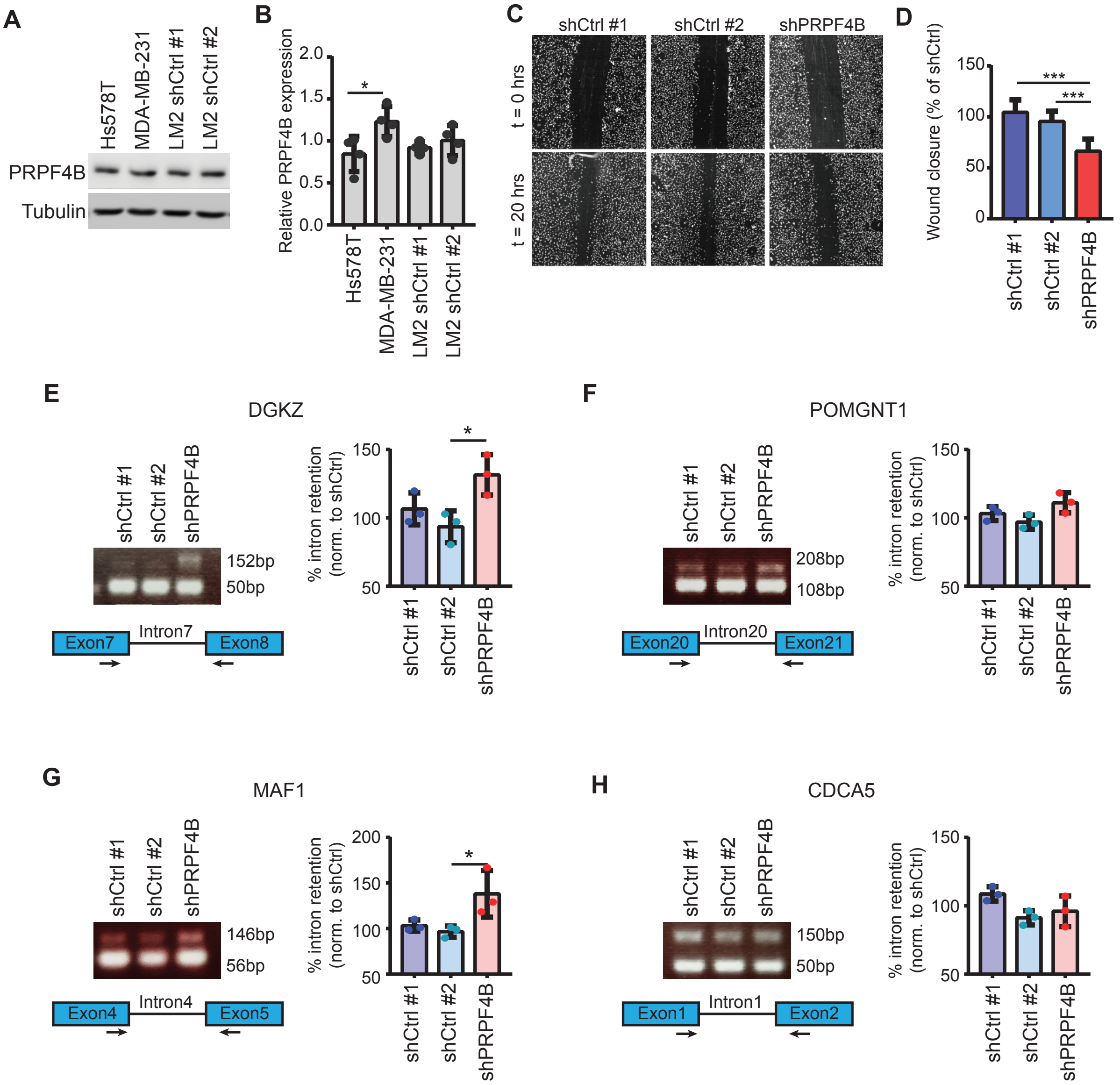

Supplementary Figure 30. ***Stable PRPF4B knockdown affects RNA splicing and cell migration.*** (A) PRPF4B expression in Hs578T, MDA-MB-231 and LM2 cell lines. (B) Quantification of G. PRPF4B expression levels were normalized to tubulin expression levels. Mean of all cell lines is equal to 1. (C) Representative images of the scratch assay in stable LM2 knockdown cell lines directly and 20 hours after making the scratch. (D) Quantification of E. Mean + stdev of 15 measurements of 2 biological replicates. DGKZ (E), POMGNT1 (F), MAF1 (G) or CDCA5 (H) intron retention in stable knockdown cell lines. Mean + stdev of 3 biological repli-cates. Significance was determined using ANOVA correcting for multiple testing. * p < 0.05, ** p < 0.01, *** p < 0.001. Source data are provided as a Source Data file.

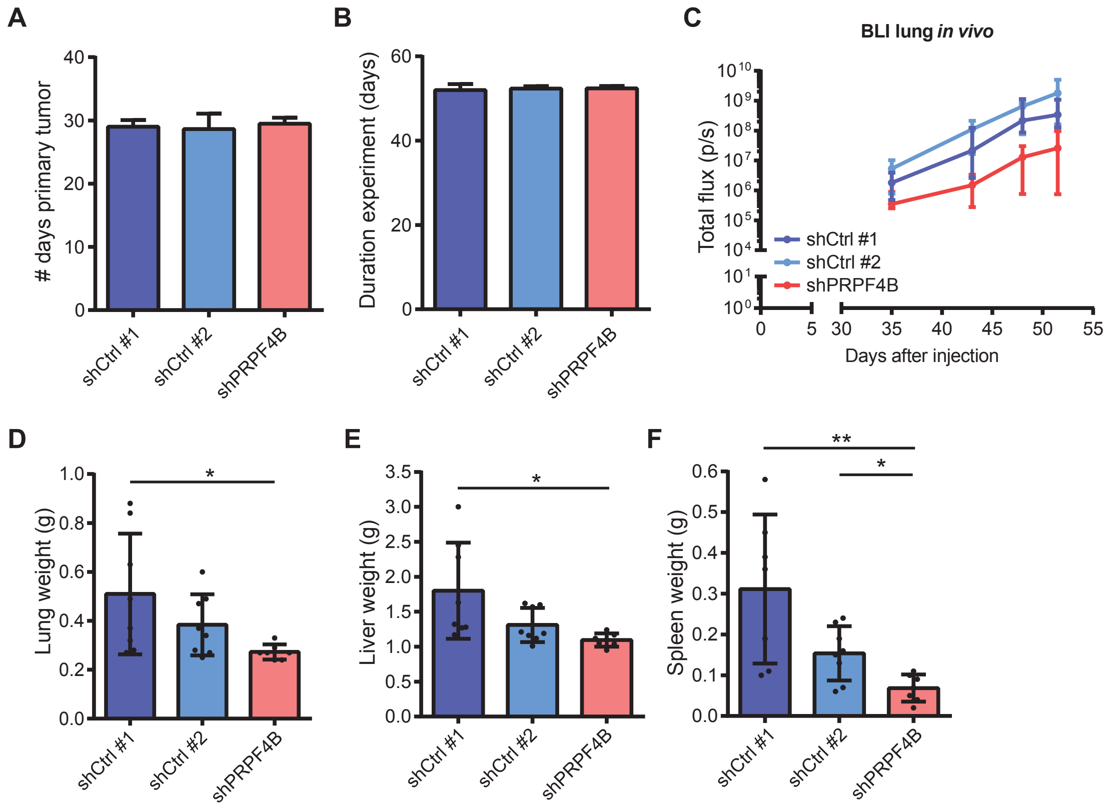

Supplementary Figure 31. ***Effect of PRPF4B downregulation on tumor growth and metastasis.*** (A) Time of removal of the primary tumor or (B) the total duration of the experiment until sacrifice. (C) Total bioluminescent flux in lungs of mice after surgery till sacrifice. Signal was normalized to lung area. Weight of the (D) lung, (E) liver and (F) spleen after sacrificing the mice. N = 7-8 mice per group. Groups were compared by Kruskal-Wallis test with Dunn's post correction test (luminescence) or ANOVA (metastasis count). * $p < 0.05$, ** $p < 0.01$. Source data are provided as a Source Data file.

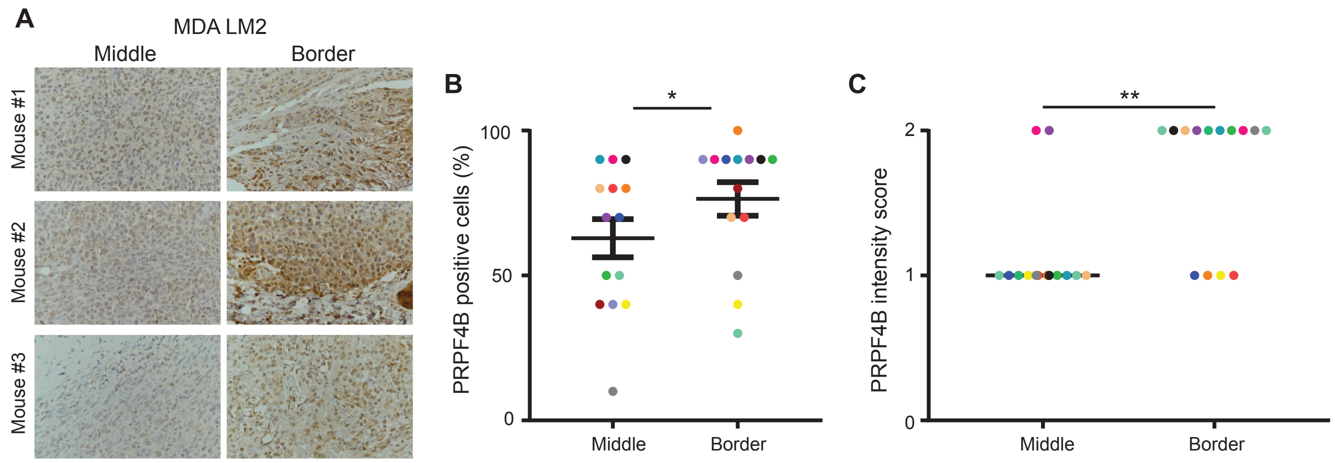

Supplementary Figure 32. ***PRPF4B expression in primary mouse tumors.*** (A) PRPF4B expression in the middle and border of primary breast tumors from of three representative mice injected with LM2 shCtrl cells. Percentage of PRPF4B positive cells (B) and PRPF4B intensity (C) in the middle and border of these tumors. Paired samples show the same color. N = 14 tumors. Significance was determined using a Wilcoxon test. * $p < 0.05$, ** $p < 0.01$. Source data are provided as a Source Data file.

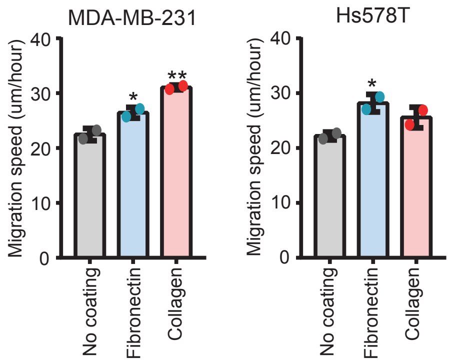

Supplementary Figure 33. ***Migration speed on different cell coatings in MDA-MB-231 and Hs578T.*** Aver-age + stdev of 2 biological replicates, significance was calculated using ANOVA with multiple testing correction. * $p < 0.05$, ** $p < 0.01$. Source data are provided as a Source Data file.

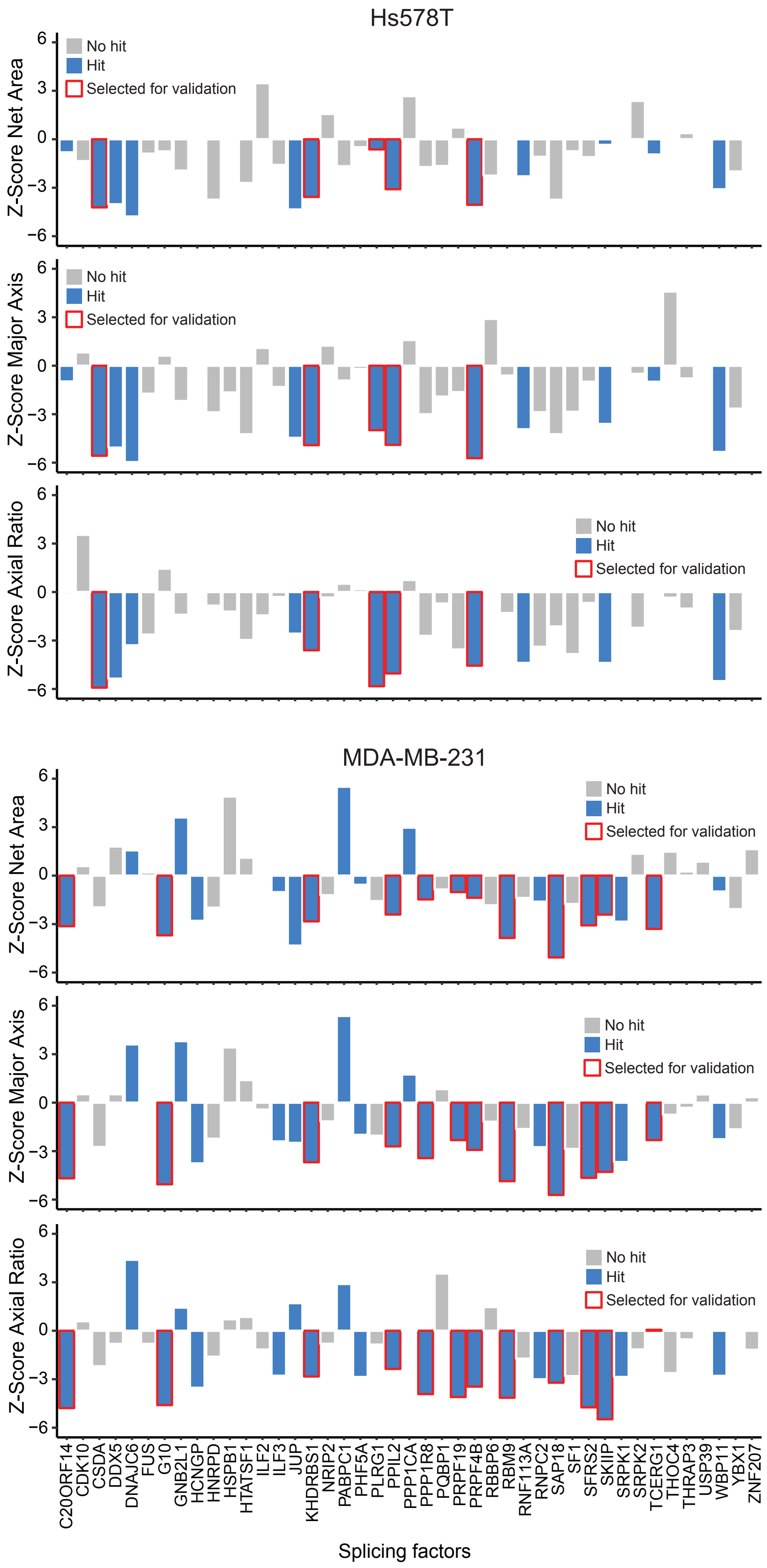

Supplementary Figure 34. ***Effect of splicing factor knockdown in the primary PKT screen in Hs578T and MDA-MB-231.*** Robust Z-scores of 2 technical and biological replicates.
