# Supplementary material for: Uncovering the signaling landscape controlling breast cancer cell migration identifies novel metastasis driver genes
Source: Nat Commun. 2019 Jul 5;10:2983. doi: 10.1038/s41467-019-11020-3 (PMC6611796; doi:10.1038/s41467-019-11020-3)
Supplement: Supplementary file 3 — Description of Additional Supplementary Files [file 41467_2019_11020_MOESM3_ESM.pdf]

Description of Additional Supplementary Files

File Name: Supplementary Movie 1
Description: Live cell imaging of MDA-MB-231 (10 frames/second, 4 minutes/frame, 10x objective, 2x2 stitching)

File Name: Supplementary Movie 2
Description: Live cell imaging of Hs578T (10 frames/second, 4 minutes/frame, 10x objective, 2x2 stitching)

File Name: Supplementary Movie 3
Description: Live cell imaging of mock control in Hs578T (10 frames/second, 12 minutes/frame, 20x objective, 2x2 stitching).

File Name: Supplementary Movie 4
Description: Live cell imaging of mock control in MDA-MB-231 (10 frames/second, 12 minutes/frame, 20x objective, 2x2 stitching).

File Name: Supplementary Movie 5
Description: Live cell imaging of siPRPF4B in Hs578T (10 frames/second, 12 minutes/frame, 20x objective, 2x2 stitching).

File Name: Supplementary Movie 6
Description: Live cell imaging of siPRPF4B in MDA-MB-231 (10 frames/second, 12 minutes/frame, 20x objective, 2x2 stitching

File Name: Supplementary Movie 7
Description: Live cell imaging of siITGB1 in Hs578T (10 frames/second, 12 minutes/frame, 20x objective, 2x2 stitching).

File Name: Supplementary Movie 8
Description: Live cell imaging of siITGB1 in MDA-MB-231 (10 frames/second, 12 minutes/frame, 20x objective, 2x2 stitching).

File Name: Supplementary Movie 9
Description: Live cell imaging of siMXD1 in Hs578T (10 frames/second, 12 minutes/frame, 20x objective, 2x2 stitching).

File Name: Supplementary Movie 10
Description: Live cell imaging of siMXD1 in MDA-MB-231 (10 frames/second, 12 minutes/frame, 20x objective, 2x2 stitching).

File Name: Supplementary Movie 11
Description: Live cell imaging of siBUD31 in Hs578T (10 frames/second, 12 minutes/frame, 20x objective, 2x2 stitching).

File Name: Supplementary Movie 12
Description: Live cell imaging of siBUD31 in MDA-MB-231 (10 frames/second, 12 minutes/frame, 20x objective, 2x2 stitching).

File Name: Supplementary Movie 13
Description: Live cell imaging of siBPTF in Hs578T (10 frames/second, 12 minutes/frame, 20x objective, 2x2 stitching).

File Name: Supplementary Movie 14
Description: Live cell imaging of siBPTF in MDA-MB-231 (10 frames/second, 12 minutes/frame, 20x objective, 2x2 stitching).

File Name: Supplementary Data 1
Description: **Primary PKT screen.** This file contains the full human drugable genome library annotation used for the primary screen, all primary PKT screen data and selected candidate geneIDs and symbols.

File Name: Supplementary Data 2
Description: **PKT screen validation.** Results of the single siRNA validation screen including validated candidates, the resulting phenotypes and number of single siRNAs confirming the smartpool results.

File Name: Supplementary Data 3
Description: **Information on cell line specific mutation of screen candidates.**

File Name: Supplementary Data 4
Description: **Live cell imaging in Hs578T.** Results of the live cell imaging in Hs578T of PKT derived candidates in Hs578T and MDA-MB-231 candidates that were significantly related to metastasis formation in the Public-344 cohort.

File Name: Supplementary Data 5
Description: **Live cell imaging in MDA-MB-231.** Results of the live cell imaging in MDA-MB-231 of PKT derived candidates in MDA-MB-231 and Hs578T candidates that were significantly related to metastasis formation in the Public-344 cohort.

File Name: Supplementary Data 6
Description: **Candidate expression related to metastasis formation in human breast cancer patients.** This file contains the PKT candidates of which the expression levels were related to metastasis-free survival in ER-negative or ER-postive tumors.

File Name: Supplementary Data 7
Description: **KEGG pathways in networks.** Results of KEGG over-representation analysis of first order PPI networks of the following datasets: candidates PKT screen Hs578T and MDA-MB-231, 440 gene Migration/Invasion signature (440 signature), Human Invastion Signature (HIS), Lung Metastasis Signature (LMS), Yu's 50 gene signature, NKI-70 signature and Wang's 76 gene signature.

File Name: Supplementary Data 8
Description: **Phenotypic screen.** Results of phenotypic screen, normalized to

File Name: Supplementary Data 9
Description: **PPI network overlap.** This file contains lists of genes in the minimum interaction PPI networks established based on candidates PKT screen Hs578T and MDA-MB-231, 440 signature, HIS, LMS, Yu's 50 gene signature, NKI-70 signature and Wang's 76 gene signature and the overlap between the different signatures.

File Name: Supplementary Data 10
Description: **Candidate expression related to metastasis formation in the Public-344 cohort.** This file contains all data relating candidate expression levels to metastasis-free survival in human BC patients using the Public-344 dataset. Stata (StataCorp) was used to perform Cox proportional hazards regression analysis, with gene expression values as continuous variable and metastasis-free survival as end point.

File Name: Supplementary Data 11
Description: **Next generation sequencing of siPRPF4B, siBUD31 and siBPTF in MDA-MB-231 and Hs578T.** This file contains basemean (mean count of control and knockdown samples), log2FC and adjusted P-values of all genes for siPRPF4B, siBUD31 and siBPTF compared to siKinasePool control. The DESeq2 package (Love, 2014) was used to normalize the data perform statistics.

File Name: Supplementary Data 12
Description: **Intron retention of siPRPF4B and siBUD31 in Hs578T and MDA-MB-231.** This file contains the intron inclusion differences and adjusted P-values of all introns for siPRPF4B and siBUD31 compared to siKinasePool.

File Name: Supplementary Data 13
Description: **Alternative splicing events of siPRPF4B and siBUD31 in Hs578T and MDA-MB-231.** This file contains the alternative 3' splice site (A3SS), alternative 5' splice site (A5SS), mutually exclusive exon (MXE) and skipped exon (SE) events and adjusted P-values for siPRPF4B and siBUD31 compared to siKinasePool.
