# Supplementary material for: Uncovering the signaling landscape controlling breast cancer cell migration identifies novel metastasis driver genes
Source: Nat Commun. 2019 Jul 5;10:2983. doi: 10.1038/s41467-019-11020-3 (PMC6611796; doi:10.1038/s41467-019-11020-3)

Figure 6E – p-FAK (Y397) – MDA-MB-231

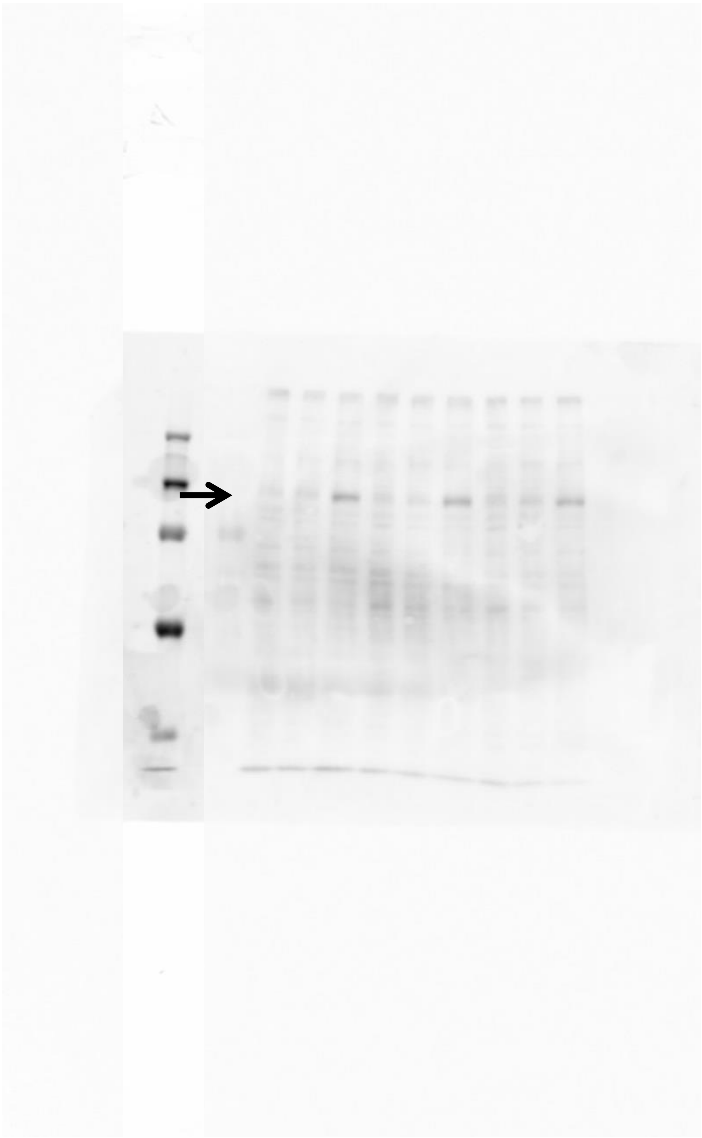

Lane #	Cell line	Knockdown
1		
2		
3	Marker	
4	MDA-MB-231	Mock
5	MDA-MB-231	siKP
6	MDA-MB-231	siBPTF
7	MDA-MB-231	Mock
8	MDA-MB-231	siKP
9	MDA-MB-231	siBUD31
10	MDA-MB-231	Mock
11	MDA-MB-231	siKP
12	MDA-MB-231	siPRPF4B
13		
14		
15		

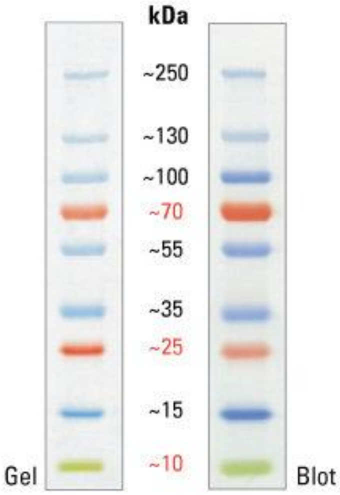

Figure 6E – FAK – MDA-MB-231

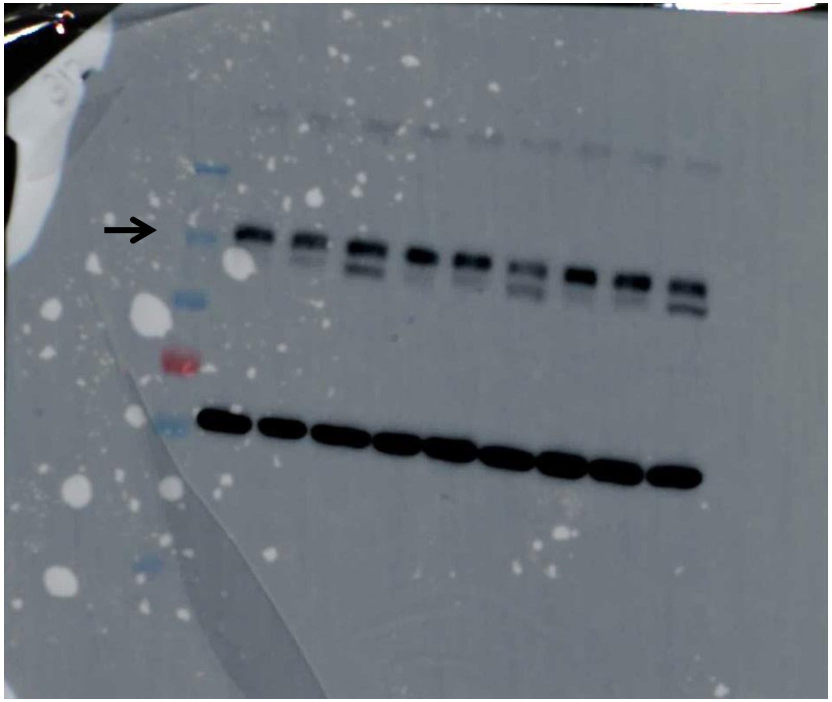

Lane #	Cell line	Knockdown
1		
2		
3	Marker	
4	MDA-MB-231	Mock
5	MDA-MB-231	siKP
6	MDA-MB-231	siBPTF
7	MDA-MB-231	Mock
8	MDA-MB-231	siKP
9	MDA-MB-231	siBUD31
10	MDA-MB-231	Mock
11	MDA-MB-231	siKP
12	MDA-MB-231	siPRPF4B
13		
14		
15		

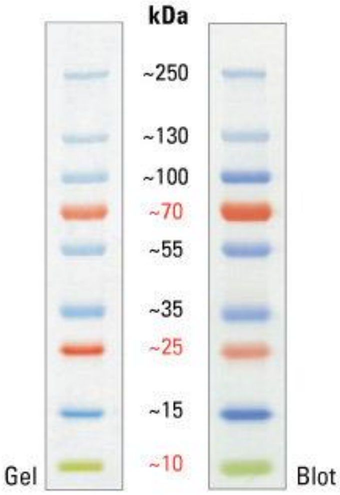

Figure 6E – ITGB1 – MDA-MB-231

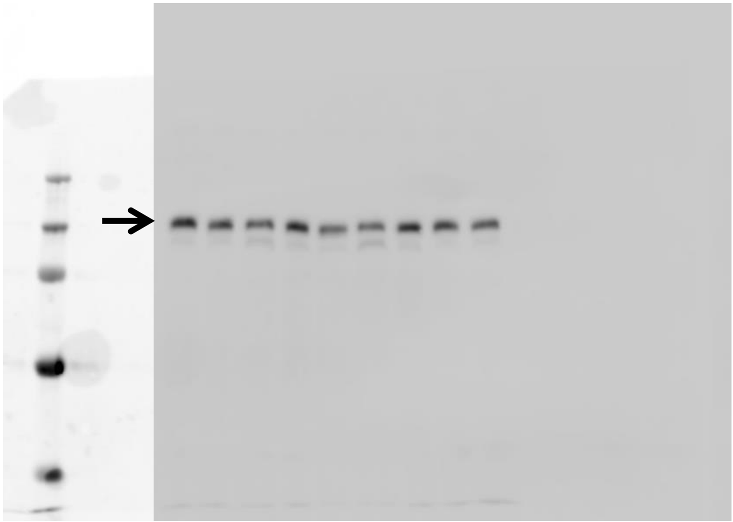

Lane #	Cell line	Knockdown
1		
2		
3	Marker	
4	MDA-MB-231	Mock
5	MDA-MB-231	siKP
6	MDA-MB-231	siBPTF
7	MDA-MB-231	Mock
8	MDA-MB-231	siKP
9	MDA-MB-231	siBUD31
10	MDA-MB-231	Mock
11	MDA-MB-231	siKP
12	MDA-MB-231	siPRPF4B
13		
14		
15		

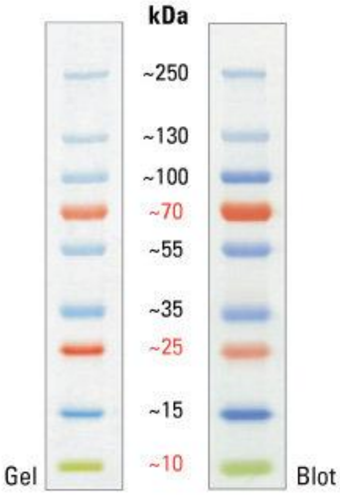

Figure 6E – PXN – MDA-MB-231

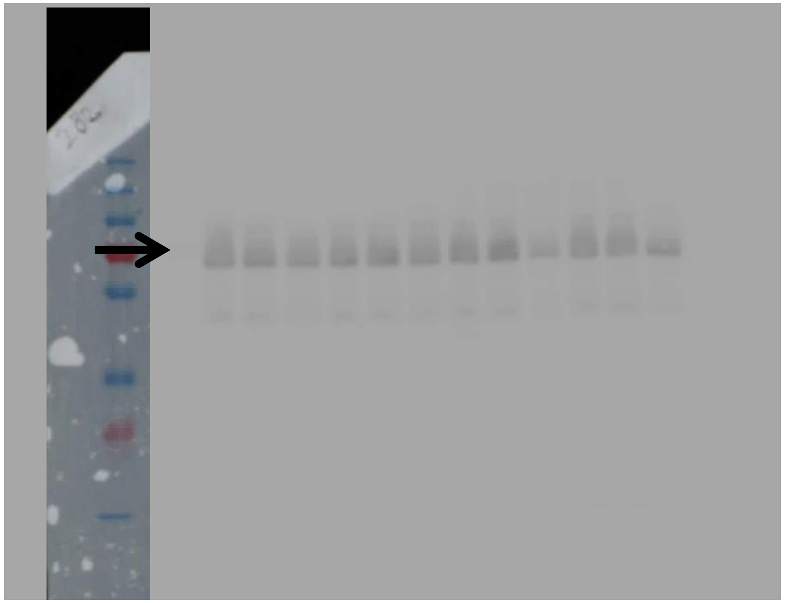

Lane #	Cell line	Knockdown
1		
2	Marker	
3	MDA-MB-231	Mock
4	MDA-MB-231	siKP
5	MDA-MB-231	siBRF1
6	MDA-MB-231	Mock
7	MDA-MB-231	siKP
8	MDA-MB-231	siBPTF
9	MDA-MB-231	Mock
10	MDA-MB-231	siKP
11	MDA-MB-231	siBUD31
12	MDA-MB-231	Mock
13	MDA-MB-231	siKP
14	MDA-MB-231	siPRPF4B
15		

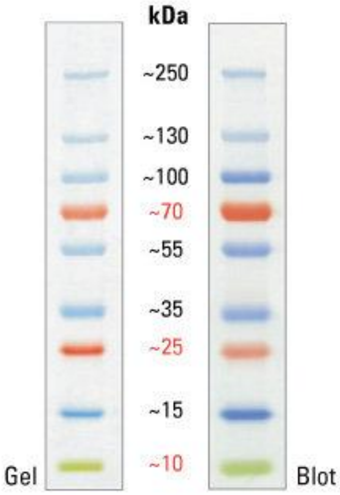

Figure 6E – ITGA3 – MDA-MB-231

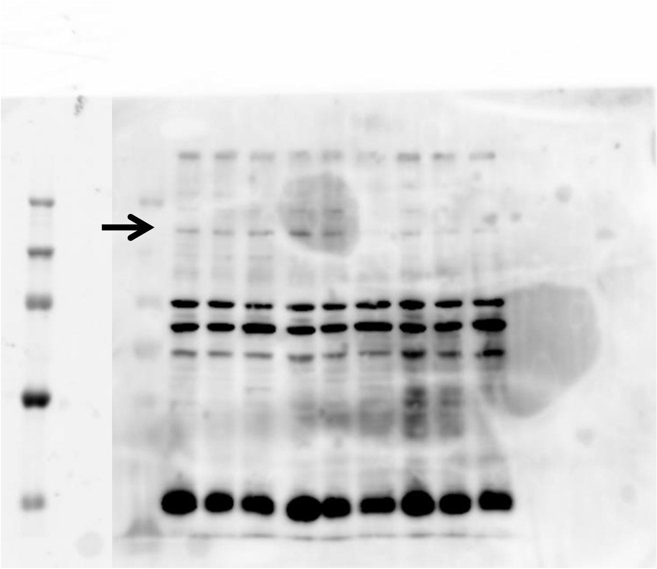

Lane #	Cell line	Knockdown
1		
2		
3	Marker	
4	MDA-MB-231	Mock
5	MDA-MB-231	siKP
6	MDA-MB-231	siBPTF
7	MDA-MB-231	Mock
8	MDA-MB-231	siKP
9	MDA-MB-231	siBUD31
10	MDA-MB-231	Mock
11	MDA-MB-231	siKP
12	MDA-MB-231	siPRPF4B
13		
14		
15		

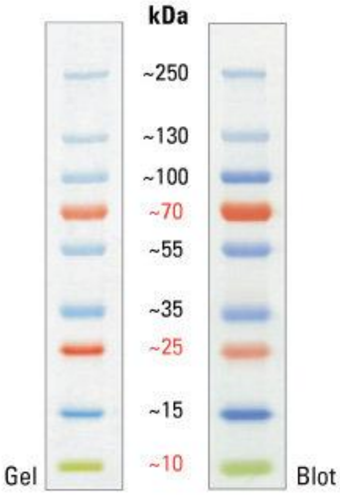

Figure 6E – LAMA5 – MDA-MB-231

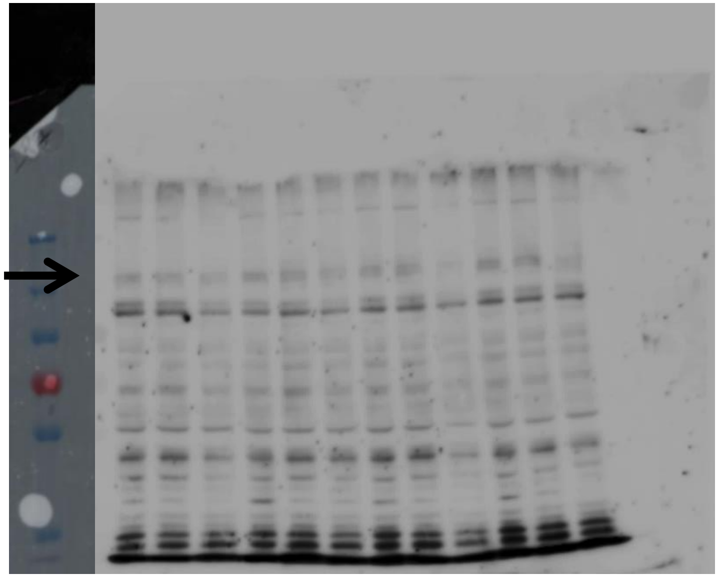

Lane #	Cell line	Knockdown
1		
2	Marker	
3	MDA-MB-231	Mock
4	MDA-MB-231	siKP
5	MDA-MB-231	siBRF1
6	MDA-MB-231	Mock
7	MDA-MB-231	siKP
8	MDA-MB-231	siBPTF
9	MDA-MB-231	Mock
10	MDA-MB-231	siKP
11	MDA-MB-231	siBUD31
12	MDA-MB-231	Mock
13	MDA-MB-231	siKP
14	MDA-MB-231	siPRPF4B
15		

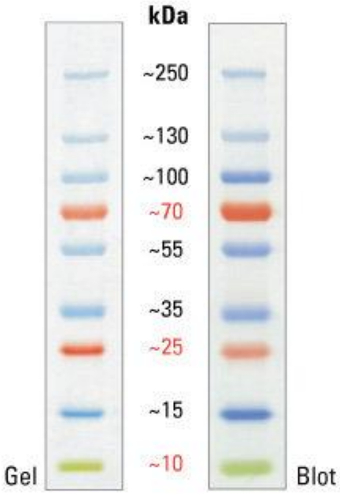

Figure 7B – PRPF4B

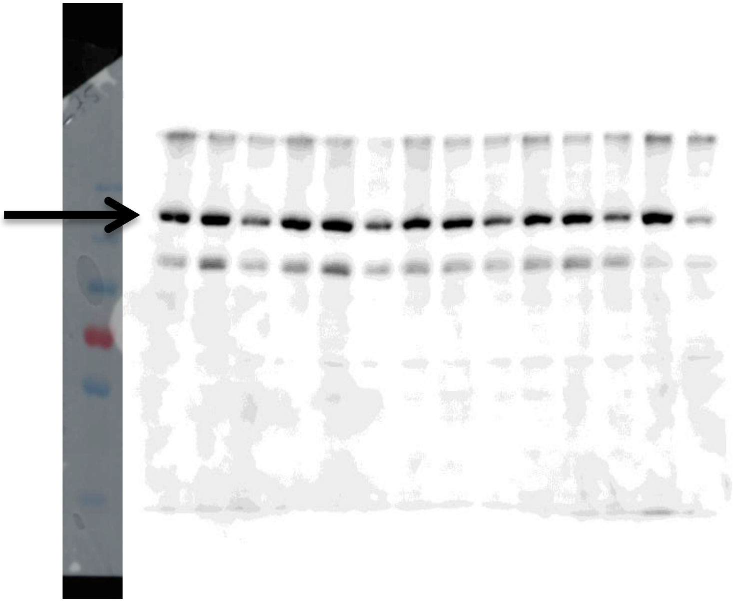

Lane #	Sample
1	Marker
2	LM2 shCtrl #1
3	LM2 shCtrl #2
4	LM2 shPRPF4B
5	LM2 shCtrl #1
6	LM2 shCtrl #2
7	LM2 shPRPF4B
8	LM2 shCtrl #1
9	LM2 shCtrl #2
10	LM2 shPRPF4B
11	LM2 shCtrl #1
12	LM2 shCtrl #2
13	LM2 shPRPF4B
14	siKinasePool
15	siPRPF4B

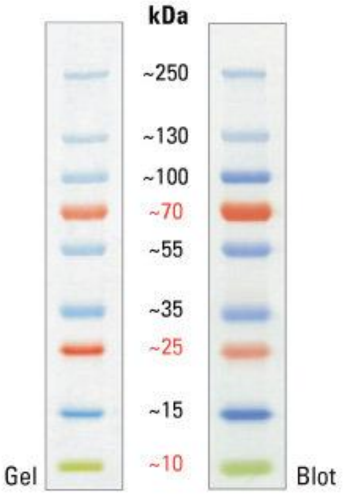

Supplementary Figure 8A – PRPF4B

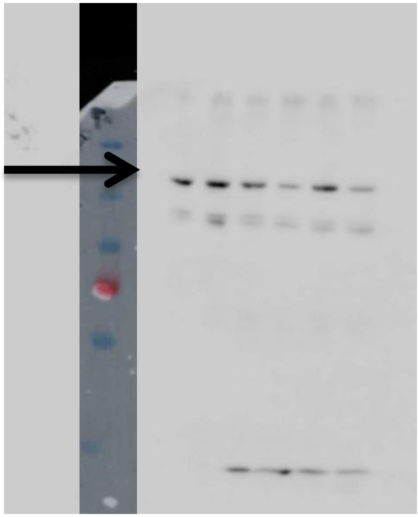

Lane #	Cell line	sgRNA	Dox treatment
1			
2	Marker	-	No
3	MDA231 WT	-	No
4	MDA231 ind Cas9	-	No
5	MDA231 ind Cas9	sgPRPF4B #1	No
6	MDA231 ind Cas9	sgPRPF4B #1	Yes
7	MDA231 ind Cas9	sgPRPF4B #2	No
8	MDA231 ind Cas9	sgPRPF4B #2	Yes
9			
10			
11			
12			
13			
14			
15			

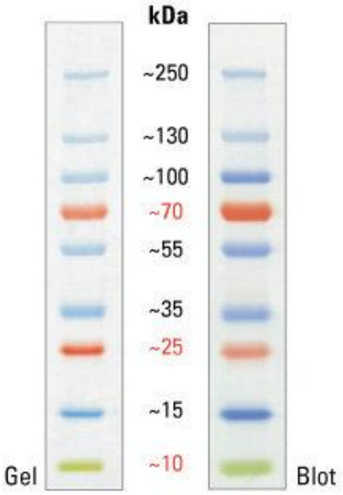

Supplementary Figure 8A – Cas9

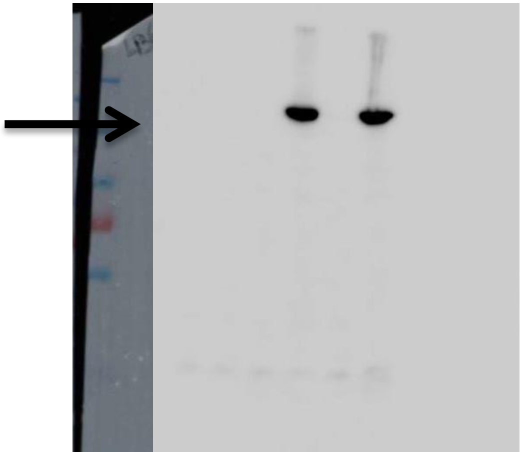

Lane #	Cell line	sgRNA	Dox treatment
1			
2	Marker	-	No
3	MDA231 WT	-	No
4	MDA231 ind Cas9	-	No
5	MDA231 ind Cas9	sgPRPF4B #1	No
6	MDA231 ind Cas9	sgPRPF4B #1	Yes
7	MDA231 ind Cas9	sgPRPF4B #2	No
8	MDA231 ind Cas9	sgPRPF4B #2	Yes
9			
10			
11			
12			
13			
14			
15			

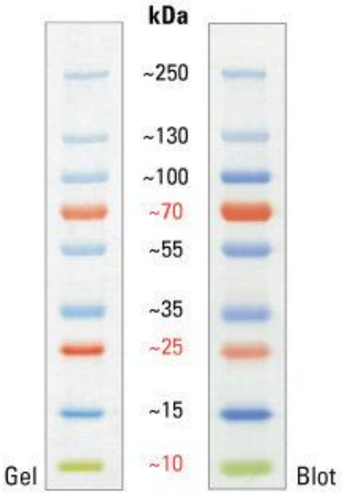

Supplementary Figure 19 – PRPF4B – MDA-MB-231

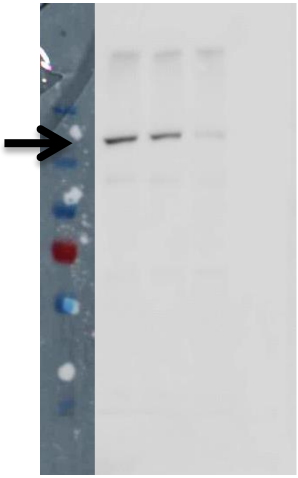

Lane #	Cell line	sgRNA
1		
2	Marker	
3	MDA231	Mock
4	MDA231	siKinasePool
5	MDA231	siPRPF4B
6		
7		
8		
9		
10		
11		
12		
13		
14		
15		

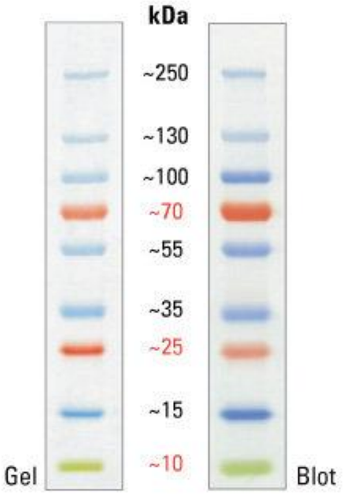

Supplementary Figure 19 – PRPF4B – Hs578T

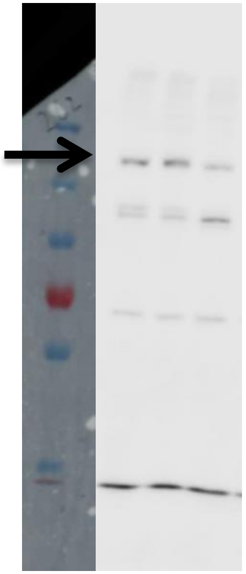

Lane #	Cell line	sgRNA
1		
2	Marker	
3	Hs578T	Mock
4	Hs578T	siKinasePool
5	Hs578T	siPRPF4B
6		
7		
8		
9		
10		
11		
12		
13		
14		
15		

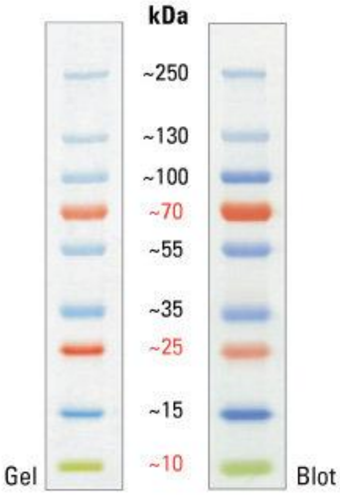

Supplementary Figure 27C – FAK (p-Y397) – Hs578T

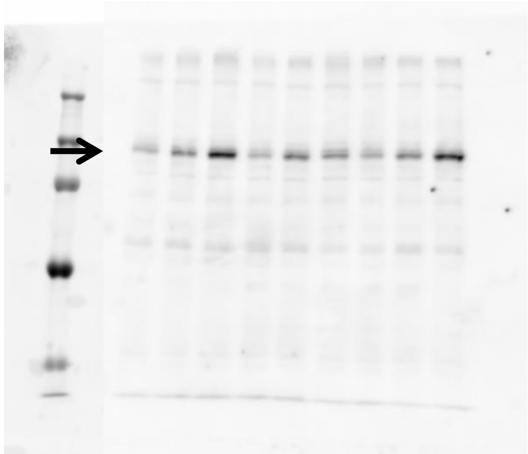

Lane #	Cell line	Knockdown
1		
2		
3	Marker	
4	Hs578T	Mock
5	Hs578T	siKP
6	Hs578T	siBPTF
7	Hs578T	Mock
8	Hs578T	siKP
9	Hs578T	siBUD31
10	Hs578T	Mock
11	Hs578T	siKP
12	Hs578T	siPRPF4B
13		
14		
15		

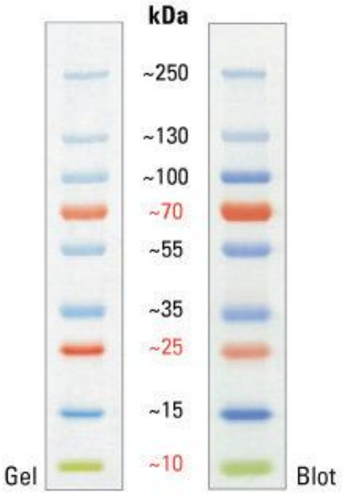

Supplementary Figure 27C – FAK – Hs578T

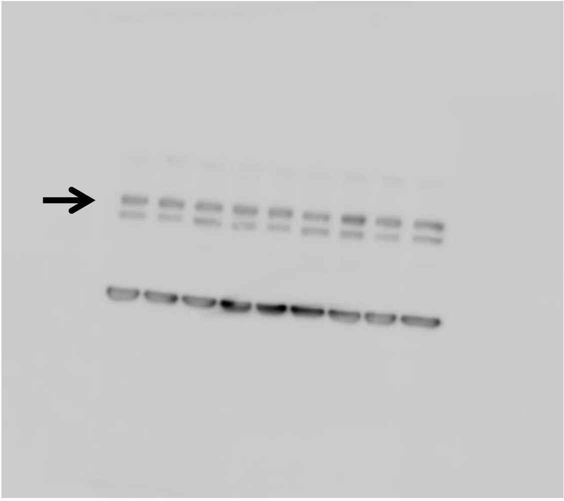

Lane #	Cell line	Knockdown
1		
2		
3	Marker	
4	Hs578T	Mock
5	Hs578T	siKP
6	Hs578T	siBPTF
7	Hs578T	Mock
8	Hs578T	siKP
9	Hs578T	siBUD31
10	Hs578T	Mock
11	Hs578T	siKP
12	Hs578T	siPRPF4B
13		
14		
15		

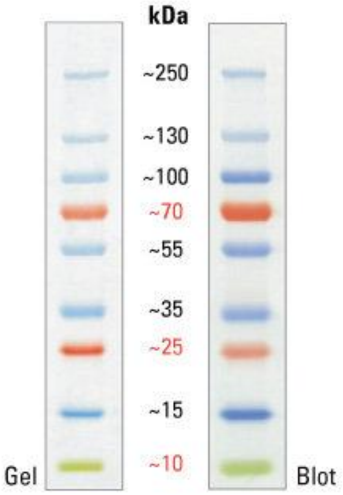

Supplementary Figure 27C – ITGB1 – Hs578T

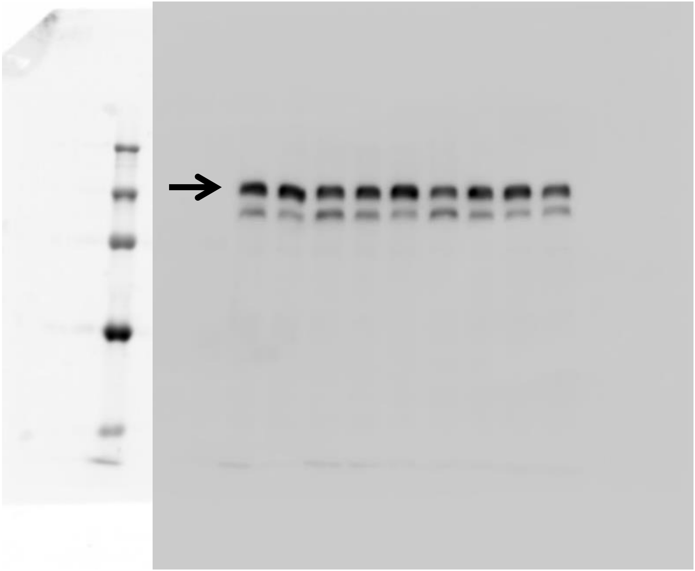

Lane #	Cell line	Knockdown
1		
2		
3	Marker	
4	Hs578T	Mock
5	Hs578T	siKP
6	Hs578T	siBPTF
7	Hs578T	Mock
8	Hs578T	siKP
9	Hs578T	siBUD31
10	Hs578T	Mock
11	Hs578T	siKP
12	Hs578T	siPRPF4B
13		
14		
15		

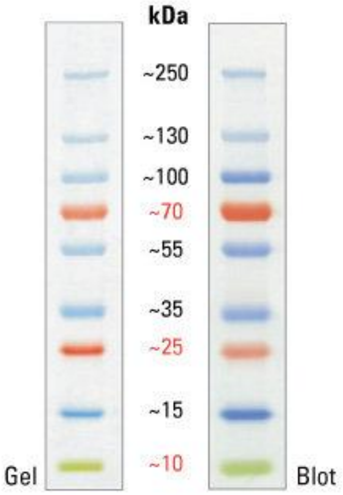

Supplementary Figure 27C – PXN – Hs578T

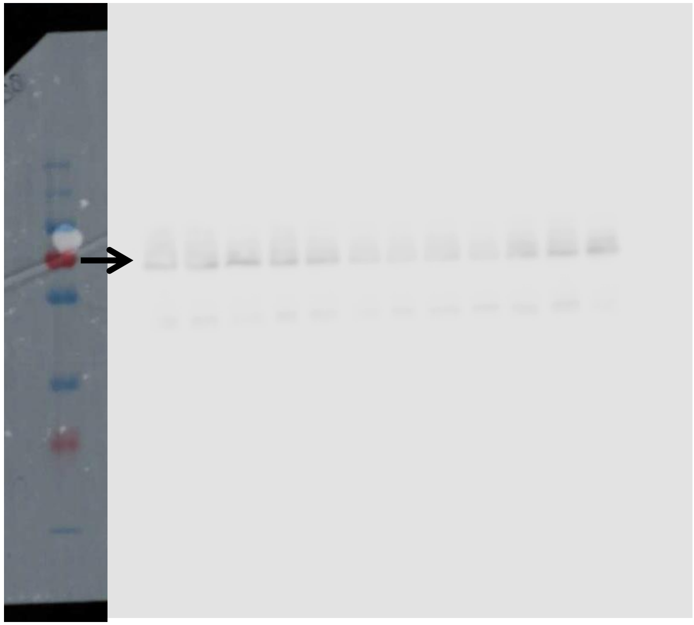

Lane #	Cell line	Knockdown
1		
2	Marker	
3	Hs578T	Mock
4	Hs578T	siKP
5	Hs578T	siBRF1
6	Hs578T	Mock
7	Hs578T	siKP
8	Hs578T	siBPTF
9	Hs578T	Mock
10	Hs578T	siKP
11	Hs578T	siBUD31
12	Hs578T	Mock
13	Hs578T	siKP
14	Hs578T	siPRPF4B
15		

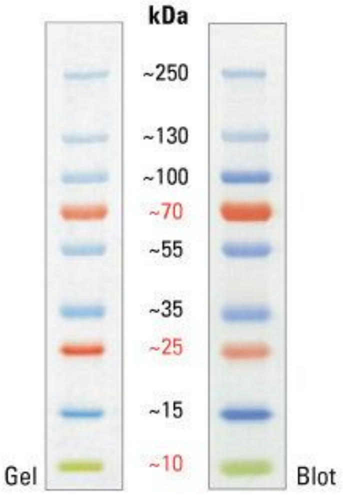

Supplementary Figure 27C – ITGA3 – Hs578T

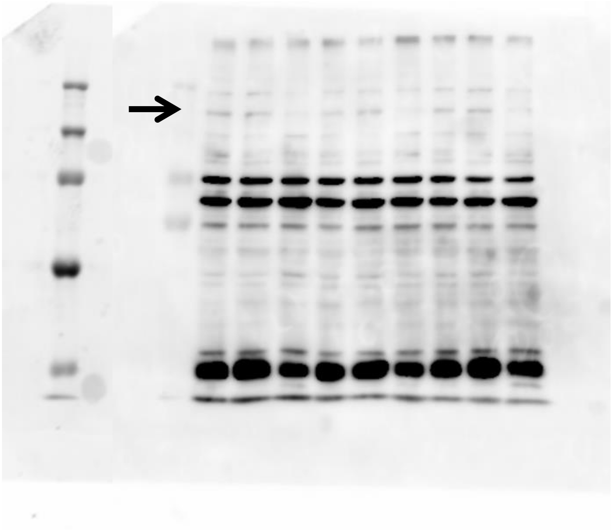

Lane #	Cell line	Knockdown
1		
2		
3	Marker	
4	Hs578T	Mock
5	Hs578T	siKP
6	Hs578T	siBPTF
7	Hs578T	Mock
8	Hs578T	siKP
9	Hs578T	siBUD31
10	Hs578T	Mock
11	Hs578T	siKP
12	Hs578T	siPRPF4B
13		
14		
15		

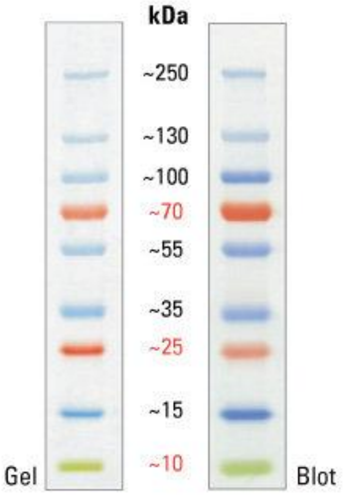

Supplement: Supplementary file 31 — Source Data [file 41467_2019_11020_MOESM31_ESM.zip › Source Data - Uncropped Western Blots.pdf]
